# Cancer Immunotherapy Based on the Bidirectional Reprogramming of the Tumor Microenvironment by a “Brakes Off/ Step on the Accelerator” Core‐Shell Manganese Phosphate/siPD‐L1 Modulator

**DOI:** 10.1002/EXP.70009

**Published:** 2025-02-09

**Authors:** Fei Xia, Yuqian Lu, Zipeng Gong, Qingchao Tu, Shuntao Liang, Chen Wang, HaiLu Yao, LinYing Zhong, Yuanfeng Fu, Pengbo Guo, Yichong Hou, Xinyu Zhou, Li Zou, Licheng Gan, Weiqi Chen, Jiawei Yan, Junzhe Zhang, Huanhuan Pang, Yuqing Meng, Qiaoli Shi, Chen Pan, Xiaomei Tao, Jigang Wang, Qingfeng Du, Chong Qiu

**Affiliations:** ^1^ State Key Laboratory for Quality Ensurance and Sustainable Use of Dao‐di Herbs, Artemisinin Research Center, and Institute of Chinese Materia Medica China Academy of Chinese Medical Sciences Beijing China; ^2^ State Key Laboratory of Discovery and Utilization of Functional Components in Traditional Chinese Medicine, Guizhou Provincial Engineering Research Center for the Development and Application of Ethnic Medicine and Traditional Chinese Medicine Guizhou Medical University Guiyang China; ^3^ State Key Laboratory of Antiviral Drugs, School of Pharmacy Henan University Kaifeng China; ^4^ BeiJing Shijitan Hospitals Capital Medical University Beijing China; ^5^ Department of Nephrology, Shenzhen key Laboratory of Kidney Diseases, and Guangdong Provincial Clinical Research Center for Geriatrics, Shenzhen Clinical Research Center for Geriatrics, Shenzhen People's Hospital (The Second Clinical Medical College, Jinan University; The First Affiliated Hospital Southern University of Science and Technology) Shenzhen Guangdong China; ^6^ Department of Traditional Chinese Medicine and School of Pharmaceutical Sciences, Guangdong Basic Research Center of Excellence for Integrated Traditional and Western Medicine for Qingzhi Diseases, Guangdong Provincial Key Laboratory of Chinese Medicine Pharmaceutics Southern Medical University Guangzhou Guangdong China

**Keywords:** cancer immunotherapy, manganese phosphate nanomodulator, nanomedicine

## Abstract

The insufficient infiltration and functional inhibition of CD8^+^ T cells due to tumor microenvironment (TME) are considered enormous obstacles to anti‐tumor immunotherapy. Herein, a pH‐responsive core‐shell manganese phosphate nanomodulator co‐loading siPD‐L1 and Mn^2+^ into nanoparticles coated with hyaluronic acid was prepared, which was aimed at the bidirectional reprogramming the tumor microenvironment: (1) “Brakes off,” restoring CD8^+^ T cells function by siPD‐L1 knockdowning PD‐L1 expression of tumor cells; (2) “Step on the accelerator,” promoting CD8^+^ T cells infiltration in tumors tissue based on the multidimensional immune effects of Mn^2+^ (immunogenic cell death induced the enhancing cGAS‐STING pathway, the proliferation and maturation of relative immune cells). Additionally, this strategy could induce macrophage polarization and inhibit the regulatory T cells in tumor site. This work provided a manganese phosphate nanomodulator to reprogram the immune TME for an enhanced comprehensive anti‐tumor effect of triple negative breast cancer, which offers a robust method for tumor immunotherapy in future clinical applications.

## Graphical abstract

To enhance the comprehensive treatment effect of triple‐negative breast cancer, a pH‐responsive core‐shell manganese phosphate nano‐regulator was prepared to reprogram the tumor microenvironment bi‐directionally. (1) “Brakes off”: Using siPD‐L1 to inhibit PD‐L1 expression to restore CD8^+^ T cell function. 2) “Step on the accelerator”: Based on Mn^2+^’s effect, promoting CD8^+^ T cell infiltration and maturation.

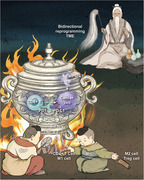

## 1 Introduction

Triple‐negative breast cancer (TNBC) has strong invasiveness, high rates of visceral and brain metastasis, and poor efficacy in endocrine therapy of hormone receptors, targeted blockade of HER2 and chemotherapy [1]. Tumor immunotherapy has made significant progress as a new treatment option for TNBC over recent years [2]. One of the key players in the immune system is the CD8^+^ T cells, which take charge of hunting down and destroying cells that have turned rogue and become cancerous. These vigilant soldiers work tirelessly to rid our bodies of any potential threats, ensuring our health and well‐being. However, inadequate infiltration and functional inhibition of CD8^+^ T cells due to tumor microenvironment (TME) have become a major obstacle to tumor immunotherapy [3]. If the tumor immunotherapy is regarded as a driving car, only when the car brake is released (“Brakes off”, restoring CD8^+^ T cells function) and the engine is refueled (“Step on the accelerator”, promoting CD8^+^ T cells infiltration), can the tumor immunotherapy car run at full speed and play a higher therapeutic effect on triple‐negative breast cancer. Thus, it stimulates a strategic need to enhance the infiltration of CD8^+^ T cells within tumors and simultaneously revive their functionality.

Immunogenic cell death (ICD) represents a hopeful strategy for activating antigen‐specific T cells and promoting their infiltration into tumors, ultimately bolstering the body's immune defenses against cancer [4]. During this process, tumor cells secrete immune stimulatory damage‐associated molecular patterns (DAMPs), including surface exposed calreticulinin (CRT), high mobility group box 1 (HMGB1) and adenosine triphosphate (ATP), which activates and promotes the maturation of dendrite cells (DCs) to increase the CD8^+^ T cells infiltration, finally transforming immune “cold tumors” into “hot tumors”. As one of the nutritional ions in the body, manganese ions (Mn^2+^) can transform the accumulated endogenous H_2_O_2_ into the strong toxic ·OH, which could trigger reactive oxygen species (ROS) storms with subsequent ICD [5]. Additionally, Mn^2+^ could greatly enhance cGAMP‐STING binding affinity, thereby activating the cGAS‐STING signaling pathway in DCs [6]. Therefore, the Mn^2+^ possesses the potential to reform the TME by mitigating hypoxia, enhancing ROS generation to activate ICDs, and promoting the proliferation and maturation of relative immune cells [7]. Numerous studies have demonstrated that exogenous addition of Mn^2+^ can effectively promote the infiltration of NK cells and CD8^+^T cells in tumor tissues, and significantly enhance the survival and amplification of memory T cells [8].

Immunosuppressive factors in the TME make the infiltrating CD8^+^T cells dysfunctional, especially the immunosuppression of immune checkpoint, such as PD‐1/PD‐L1and so on [9]. Using serial immune checkpoint inhibitors to relieve immunosuppression is an important choice for TNBC treatment. For example, the preliminary anti‐tumor activity results of chemotherapy combined with PD‐1 or PD‐L1 inhibitor in the neoadjuvant treatment of TNBC showed significant anti‐tumor activity in patients [10]. Among them, PD‐L1 siRNA (siPD‐L1) can serve as the gene silencing tool to knockdown the expression of PD‐L1 in cancer cells specifically [11], resulting in the enhanced anti‐tumor immune response to kill cancer cells by preventing the interaction between PD‐L1 and PD‐1 on T cells. However, siRNA has some defects, such as electronegativity, easy to be destroyed by nucleases, difficulty to be taken up by cells, and intracellular lysosomal degradation [12]. Nano delivery system has become a common strategy to solve the drug formation obstacles, such as liposomes, micelles, nanocomposites and so on [13]. Additionally, metal ions (such as Ca^2+^, Zn^2+^, and Mn^2+^) can coordinate with the phosphate group of nucleic acid backbone in gene drugs (DNA or siRNA, etc.) to form a nanoprecipitation core, whose outer layer is coated with lipid materials, PEG and hyaluronic acid to achieve the purpose of encapsulated delivery [14]. This nanocarrier has the following advantages: (1) drugs are encapsulated in the core region to avoid leakage and degradation; (2) pH‐responsive property induces the release of drugs under the acidic environment of lysosomes and the quick‐increased osmotic pressure can promote the lysosomal escape; (3) most of the assembly materials are in vivo components or biodegradable polymers with good biocompatibility.

**SCHEME 1:**
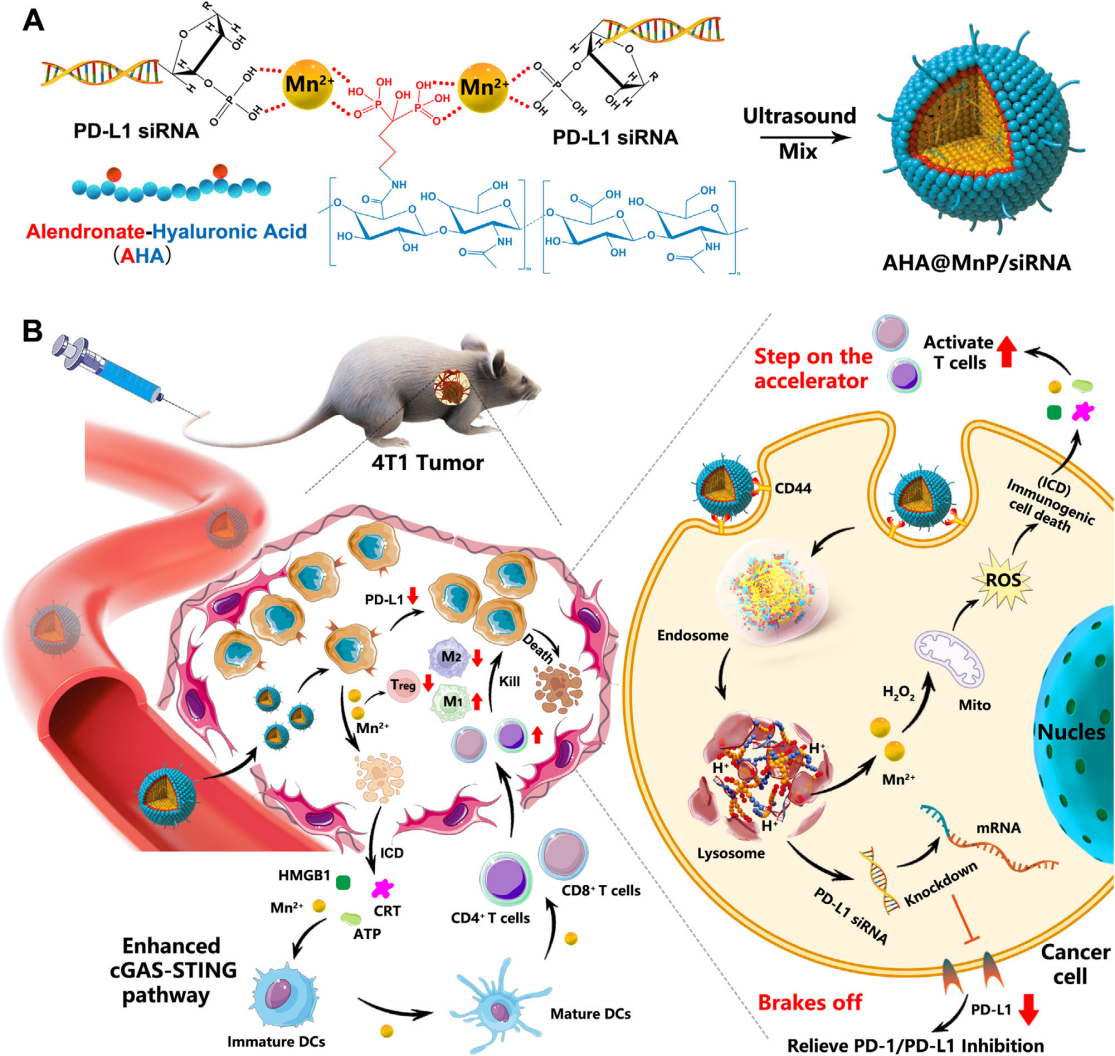
Schematic diagram of core‐shell manganese phosphate nanomodulator enhances anti‐tumor immunotherapy via bidirectional reprogramming of the tumor microenvironment. (A) Assembly of AHA@MnP/siPD‐L1 NPs; (B) The “Brakes off/Step on the accelerator” strategy of bidirectional reprogramming of the tumor immune microenvironment to exert the effect of combined immunotherapy: (1) overloaded Mn^2+^ in the cytosol induce ICD to release DAMPs and Mn^2+^ to activate the cGAS‐STING pathway of DCs, thereby inducing the proliferation and maturation of immune cells and promoting CD8^+^ T cells infiltration in tumors tissue; (2) siPD‐L1 knockdown the PD‐L1 expression of tumor cells to restore the CD8^+^ T cell function.

Herein, based on the “Brakes off/Step on the accelerator” strategy of bidirectional reprogramming of the tumor immune microenvironment, we prepared a pH‐responsive nanomodulator (AHA@MnP/siPD‐L1 nanoplexes) by coloading siPD‐L1 and Mn^2+^ into nanoparticles coated with hyaluronic acid (HA) based on the coordination of Mn^2+^ with the phosphate backbone of alendronate and siPD‐L1 (Scheme 1A). Compared to existing manganese‐based nanoparticles such as ultrasmall PtMn nanoparticles (PtMn) [15], selenium‐doped manganese phosphate nanoparticles (OX@Se‐MnP) [16], and cisplatin prodrug‐loaded manganese‐deposited iron oxide nanoplatform (Pt‐FMO) [17], the nanoparticles have a simpler synthesis strategy, enhanced pH sensitivity and targeting, and better biocompatibility, which contribute to further clinical development applications.

As shown in Scheme 1B, first, the negative hyaluronic acid can prolong the circulation time of NPs in vivo and accumulate in the tumor site through the enhanced permeability and retention (EPR) effect, followed by a higher cellular uptake based on the receptor‐ligand action between hyaluronic acid and CD44 which is overexpressed on the cell membrane of TNBC. Second, in the lysosomal acidic environment, the pH‐responsive dissolution of manganese phosphate nanoplexes will induce the osmotic effect to promote lysosomal escape and release drug molecules. Finally, the bidirectional reprogramming of the TME exert the synthesized effect of immunotherapy: (1) overloaded Mn^2+^ in the cytosol induce ICD to release DAMPs and Mn^2+^ to activate DCs, thereby inducing the proliferation and maturation of immune cells, and promoting CD8^+^ T cells infiltration in tumors tissue; (2) siPD‐L1 knockdown the PD‐L1 expression to restore the killing function of CD8^+^ T cell. These in vitro and in vivo results suggested that the manganese phosphate nanomodulator (AHA@MnP/siPD‐L1 NPs) would be an important and effective strategy to enhance the antitumor immune response in poorly immunogenic tumors, presenting new opportunities to improve the efficacy of immunotherapy in clinical application.

## 2 Results and Discussion

### 2.1 Preparation and Characterization of AHA@MnP/siPD‐L1 NPs

The preparation of AHA@MnP/siRNA NPs was outlined in Scheme 1A, with detailed information provided in the materials and methods section of the Supporting Information based on our prior research [14a]. First, the alendronate‐hyaluronan graft polymers (AHA) were synthesized by the procedure in Figure S1. The yield rate of alendronate was approximately 65% based on the integral area ratio of corresponding active hydrogen ([*a* + *b*]/2*c*) in ^1^H‐NMR spectrum. Then, MnCl_2_ solution (containing siRNA) was dropped into the HEPES buffer containing different concentrate of AHA under ultrasonification to assemble the core‐shell nanoparticles. Based on the coordination of Mn^2+^ with the phosphate backbone of alendronate and siPD‐L1, siRNA and Mn^2+^ were loaded into the inner core with an outer shell of hyaluronic acid. The effects of different concentrations of AHA and Mn^2+^ on nanoparticle size, polydispersity index (PDI) and encapsulation efficiency were explored, and the preferred AHA@MnP/siRNA NPs were prepared under the concentration of AHA and Mn^2+^ was 3.0 mg mL^−1^ and 100 mM, respectively (Figure S2A–C).

**FIGURE 1:**
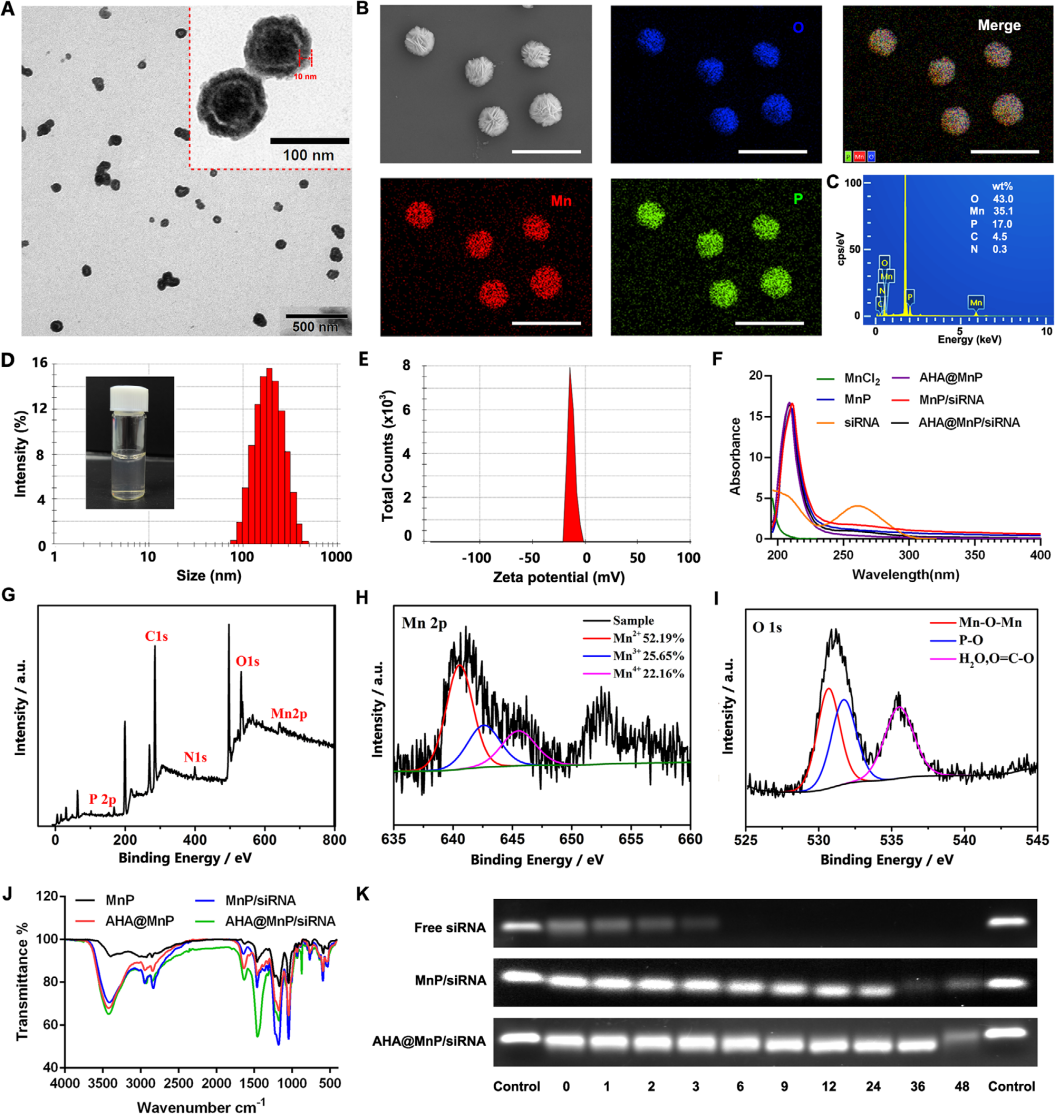
Structural characterization of AHA@MnP/siPD‐L1 NPs. (A) TEM of AHA@MnP/siPD‐L1 NPs. (B) Scanning electron microscopic (SEM) and EDS image of AHA@MnP/siPD‐L1 NPs. The scale bar is 500 nm. (C) EDS image of AHA@MnP/siPD‐L1 NPs. (D) DLS analysis on the size distribution of AHA@MnP/siPD‐L1 NPs. (E) DLS analysis of the zeta potential of AHA@MnP/siPD‐L1 NPs. (F) UV–vis spectra of nanoparticles. (G) XPS analysis of AHA@MnP/siPD‐L1 NPs, (H) Mn 2p and (I) O 1s peaks. (J) FT‐IR analysis of nanoparticles. (K) Gel retardation assay of siRNA (final concentration of 1 µM).

The morphologies, particle sizes and zeta‐potentials of as‐prepared NPs were first determined by the transmission electron microscope (TEM), scanning electron microscopy (SEM) and dynamic light scattering (DLS), respectively. These results revealed that the AHA@MnP/siRNA NPs exhibited a uniform spherical core‐shell nanostructure with a particle diameter near 110 nm, and the winding filament‐like shell (≈10 nm) tended to the nanosheets structure (Figure 1A–B), which was smaller than the hydrated diameter (196.13 ± 0.55 nm) detected by DLS (Figure 1D and Table S1). Compared to the uncoated MnP/siRNA NPs which tended to the sedimentation (1899.67 ± 79.20 nm) (Figure S3 and Table S1), AHA@MnP/siRNA NPs showed a smaller uniform diameter with clear and purplish red because the rapidly growing manganese phosphate siRNA co‐precipitate was stabilized by the AHA shell. However, it possessed a similar siRNA encapsulation efficiency (91.3 ± 2.99%) with obvious aggregated MnP/siRNA NPs (89.2 ± 1.34%) (Figure S4). Meanwhile, the zeta‐potentials of AHA@MnP/siRNA NPs were −17.25 ± 1.01 mV due to the negative AHA shell (Figure 1E and Table S1), which contributed to the safety of in vivo applications.

Furthermore, the composition and structural composition were analyzed through energy‐dispersive spectroscopy (EDS), X‐ray photoelectron spectroscopy (XPS), UV–vis–NIR absorption spectra and Fourier Transform Infrared (FT‐IR) analysis. Figure 1B–C shows the SEM image and elemental mappings of NPs, indicating the elemental signals of Mn, P, O, C and N based on the EDS analysis. These were further confirmed by XPS analysis (Figure 1G–I), which demonstrated that the NPs contained P (132.96 eV), C (284.77 eV), N (399.45 eV), O (531.99 eV), Mn (641.96 eV) elements (Figure 1G). As displayed in Figure 1H, the Mn 2p high‐resolution XPS spectrum contains two energy bands, which can be further divided into three components: Mn^2+^ (52.19%), Mn^3+^ (25.65%) and Mn^4+^(22.16%). This might be because that Mn^2+^ was oxidized by air to Mn^3+^ or Mn^4+^ during the preparation process (pH 7.4 – 7.6, a weakly alkaline condition). Similarly, the O 1s spectrum exhibits peaks at 530.6, 531.7, and 535.5 eV attributed to the metal‐oxygen bond (Mn─O─Mn), P─O and surface oxygen (H_2_O, O═C─O) (Figure 1I). The XPS analysis applied to MnP/siPD‐L1 NPs and AHA@MnP NPs showed a similar binding energy of Mn 2p, O 1s, P 2p, N 1s and C 1s (Figure S5). Besides, UV–vis–NIR absorption spectra analysis indicated that MnP, MnP/siRNA, AHA@MnP and AHA@MnP/siRNA NPs showed an iconic absorption peak at 210 nm (Mn). Otherwise, siRNA, MnP/siRNA and AHA@MnP/siRNA NPs showed an iconic absorption peak at 260 nm (siRNA) (Figure 1F). As the Fourier transform infrared (FT‐IR) analysis shown in Figure 1J, the bending vibration of OH could be attributed to peaks at 3422, 2923 and 2850 cm^−1^, and the bending vibration of C═O could be attributed to peaks at 1630 cm^−1^, while those at 1458 cm^−1^ could be ascribed to the stretching vibration of NH. Based on this elemental and compositional analysis. Taken together, the abovementioned characterization results suggest the successful synthesis of AHA@MnP/siRNA NPs.

To evaluate the stability of AHA@MnP/siRNA NPs, changes in particle size under various dilution factors, different media, and long‐term storage were analyzed using DLS. These findings revealed that the NPs demonstrated a significant anti‐dilution effect (≈500 times, Figure S2D) and maintained a consistent size distribution in diverse media (Figure S2E). Moreover, the NPs exhibited good stability when stored at room temperature for up to one week (Figure S2F). Furthermore, the protective effects against RNase on siRNA were assessed through the gel retardation assay. As depicted in Figure 1K, compared to naked siRNA (≈3 h) and MnP/siRNA NPs (≈24 h), AHA@MnP/siRNA NPs (≈48 h) exhibited superior protection against RNase degradation in serum due to the outer HA shell. Overall, these results indicated that AHA@MnP/siRNA NPs possessed stability in blood circulation and showed the potential for in vivo applications.

### 2.2 TME Bidirectional Reprogramming Capacity of AHA@MnP/siRNA NPs In Vitro

The immunosuppressive and therapeutic‐resistant nature of the TME has been attributed to insufficient infiltration and the functional inhibition of CD8^+^ T cells [3a–c]. The functional killing of CD8^+^ T cells is often inhibited by various factors, mainly including the lower pH, hypoxia, and high levels of hydrogen peroxide (H_2_O_2_) environment and the immunosuppression of immune checkpoint (such as PD‐1/PD‐L1). We assumed that AHA@MnP/siRNA NPs could reform the TME by a bidirectional reprogramming capacity. On one hand, MnP precipitation consisting of Mn^2+^, Mn^3+^ and Mn^4+^ can react with protons to elevate the pH and serve as a nanozyme to alleviate hypoxia by breaking down intracellular H_2_O_2_, leading to a reduction in acidity and high levels of H_2_O_2_, respectively. Additionally, siPD‐L1 can knockdown the PD‐L1 expression to release the inhibition of immune checkpoint of PD‐1/PD‐L1 [18]. These activities contribute to a combined “Brakes off” effect on immune therapy. On the other hand, the MnP precipitation contributes to the generation of ROS, resulting in the ICD apoptosis for the immune cell polarization and activation, which is like “Step on the accelerator” of immune therapy. Thus, AHA@MnP/siPD‐L1 NPs have the potential to enhance the anti‐tumor effects under the “Brakes off/Step on the accelerator” strategy synergistically.

#### 2.2.1 The “Brakes Off” Effect of AHA@MnP/siPD‐L1 NPs In Vitro

**FIGURE 2:**
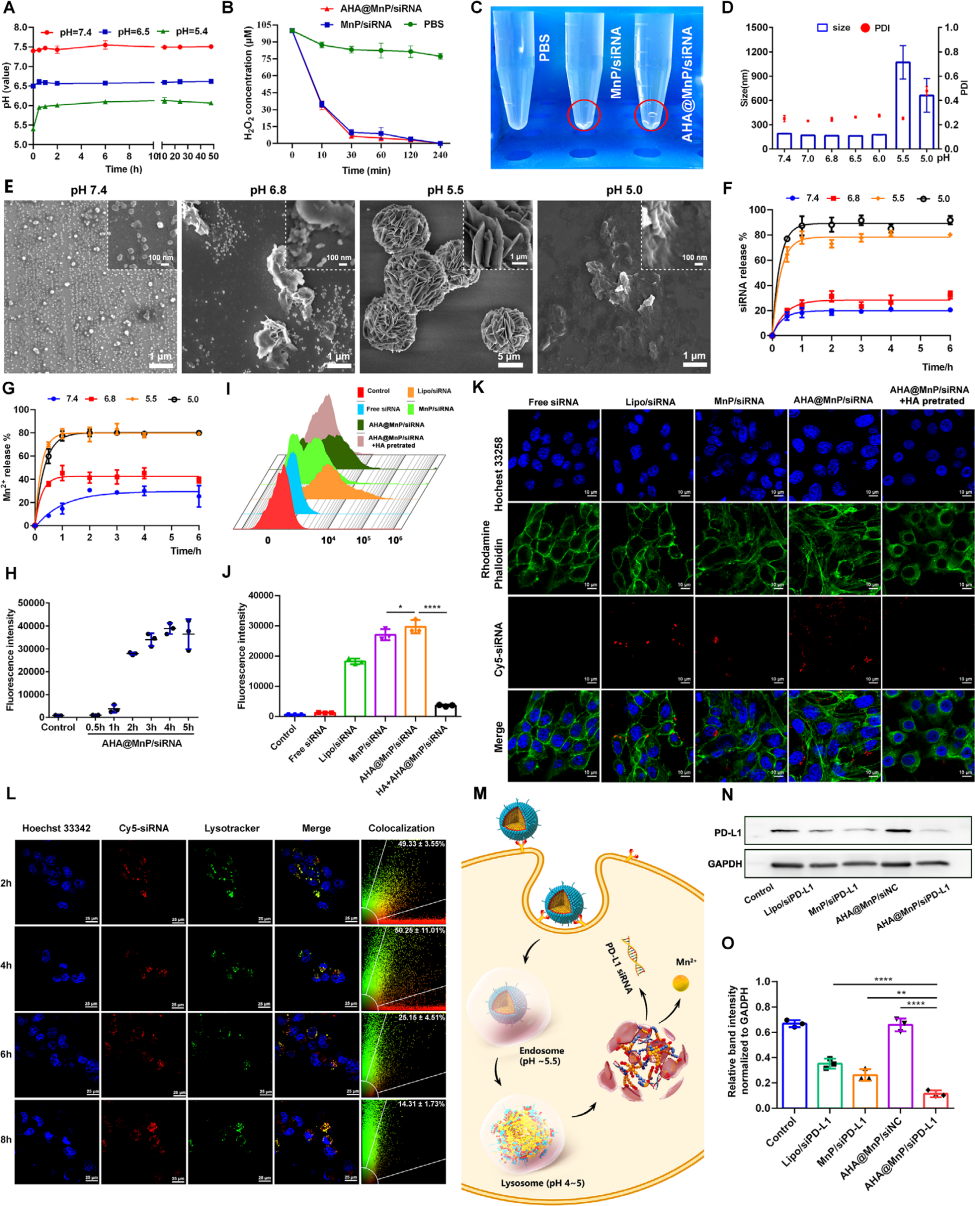
The “Brakes off” effect of AHA@MnP/siPD‐L1 NPs in vitro and in cancer cells. (A) Acidity neutralization profiles of NPs. (B) Curve of H_2_O_2_ decomposition. (C) Image of H_2_O_2_ decomposition after treatment with different NPs (red circle, gas bubble). (D) DLS analysis on the size distribution of AHA@MnP/siPD‐L1 NPs in Opti‐MEM medium at different pH conditions. (E) TEM of AHA@MnP/siPD‐L1 NPs in Opti‐MEM medium solutions with different pH. (F) siRNA and (G) Mn^2+^ release of AHA@MnP/siPD‐L1 NPs in different pH HEPES solutions. (H–J) Quantitative analysis of intracellular fluorescence intensities by flow cytometry (FAM‐labeled siRNA: 100 nM, *n* = 3). (K) The intracellular fluorescence intensities detected by confocal microscopy. (L) Confocal imaging of co‐localization of AHA@MnP/siPD‐L1 NPs (Cy5‐labeled siPD‐L1, red) with lysosomes (LysoTracker green, green) in 4T1 cells. Hoechst 33258 (blue) was used for staining the nucleus. (M) The schematic illustration of lysosomal escape of siRNA and Mn^2+^ in a pH‐dependent manner. (N) and (O) The expression of the PD‐L1 protein detected by the western‐blot assay. The data are shown as mean ± SD (*n* = 3). **p < 0.01 and ****p < 0.0001.

The study initially focused on the bidirectional reprogramming potential of AHA@MnP/siPD‐L1 NPs in the TME. The AHA@MnP/siPD‐L1 NPs were suspended in HEPES buffer solutions at varying pH levels (7.4 (physiological environment), 6.5 (TME), and 5.4 (acidic environment in endosome or lysosome)), and the resulting pH changes were monitored. As shown in Figure 2A, the pH values of 7.4, 6.5, and 5.4 were significantly elevated to 7.51, 6.63, and 6.13 within 0.5 h, respectively, showcasing the remarkable acidity‐downregulation ability of the NPs. The AHA@MnP/siPD‐L1 NPs might increase the pH value mainly via the following steps: (Mn)_3_(PO_4_)_2_ (s) + H^+^ ↔ Mn^2+^ + H_2_PO_4_^−^, MnHPO_4_ (s) + H^+^ ↔ Mn^2+^ + H_2_PO_4_^−^ and MnPO_4_ (s) + H^+^ ↔ Mn^3+^ + H_2_PO_4_^−^ (and/or HPO_4_^2−^) and MnO(OH) (s) + H^+^ ↔ Mn^3+^+ H_2_O. Subsequently, the decomposition of H_2_O_2_ by AHA@MnP/siRNA NPs was assessed by introducing NPs into a solution containing H_2_O_2_. The quantitative analysis results demonstrated that both AHA@MnP/siRNA NPs and MnP/siRNA NPs effectively and rapidly decomposed H_2_O_2_ in vitro. Following a 4 h incubation period, complete decomposition of H_2_O_2_ was observed (Figure 2B) and the appearance of bubbles (red circle) indicated the generation of oxygen (O_2_) (Figure 2C), which might mainly via the following step: Mn^3+^ + H_2_O_2_ ↔ Mn^2+^ + H_2_O + O_2_ and MnO_2_ + H_2_O_2_ ↔ Mn^2+^ + H_2_O + O_2_. To explore this mechanism, we performed bulk RNA‐seq analysis of AHA@MnP/siPD‐L1 NPs‐treated mouse tumors and found that it caused a significant downregulation of genes related to anaerobic energy metabolism (Figure S6A). As shown in Figure S6B–C, treatment with MnP/siPD‐L1 NPs and AHA@MnP/siPD‐L1 NPs resulted in a significant down‐regulation of Eno1 expression, indicating a significant decrease in intracellular glycolysis, a metabolic change that was attributed to increased oxygen in the tumor microenvironment [19]. These results suggested that the generation of oxygen (O_2_) derived from AHA@MnP/siRNA NPs had the potential to alleviate hypoxia within the TME [20].

To investigate the pH sensitivity of NPs, we analyzed the diameter and morphology of the AHA@MnP/siRNA NPs in Opti‐MEM medium solutions with pH values ranging from 5.0 to 7.4 at 25°C. As shown in Figure 2D, when the extracellular pH value is greater than 6.0, there is no significant change in particle size as pH decreases. However, when the intracellular pH value is less than 6.0, the particle size gradually increases as pH decreases, leading to a significant increase in the polydispersity index (PDI). Additionally, changes in morphology were observed using TEM (Figure 2E). It is evident that as pH decreases, nanoparticles gradually expand and loosen into sheets at pH 5.5, eventually dissolving into smaller units at pH 5.0. The AHA@MnP/siRNA NPs exhibit pH‐sensitive disassembly in weakly acidic conditions (pH 5.0–5.5) of endosomes or lysosomes, facilitating to release siRNA and Mn^2+^ into the cytosol for further treatment. The release kinetics of siRNA and Mn^2+^ from the nanoparticles were monitored using Quant‐iT RiboGreen RNA Reagent Kit and spectrophotometrically potassium periodate, respectively. As shown in Figure 2F, release profiles demonstrate that siRNA was well maintained at a near‐neutral physiological environment (pH 7.4) and TME (pH 6.8), with final release ratios of around 19.7% and 29.6% after 6 h, respectively. In contrast, a significant increase in release was observed at endosomal and lysosomal pH levels of 5.5 and 5.0, with final release ratios up to 80.3% and 94.1%, respectively. Similarly, Mn^2+^ release was triggered rapidly at lower pH levels, with around 80% released within 1 h (Figure 2G), validating the quick drug release ability of AHA@MnP/siRNA NPs in endosomes or lysosomes [21].

Furthermore, the cellular uptake of nanoparticles in 4T1 cells was first measured by flow cytometry. The intracellular fluorescence intensity of AHA@MnP/siRNA NPs gradually increased over time (Figure 2H) and could obtain a similar effect at different concentrate of AHA in NPs (Figure S7). The intensity was notably higher compared to both MnP/siRNA NPs and siRNA groups, as well as the Lipo/siRNA group (a positive control) after a 4 h incubation (Figure 2I–J). Meaningfully, after pretreating with free hyaluronic acid (HA), there was a sharp decrease in the uptake of AHA@MnP/siRNA NPs. The phenomenon was consistent with the Cy5‐siRNA (red) in cancer cells detected by the confocal microscopy that the AHA@MnP/siRNA NPs possessed more red dots in 4T1 cells (Figure 2K). The observed decrease in uptake after pre‐treatment with free hyaluronic acid (HA) could be attributed to HA's role as a natural mucopolysaccharide known to act as a targeting ligand for the CD44 membrane protein, which is often overexpressed in 4T1 cells [22]. These findings implied that AHA@MnP/siRNA NPs might enhance cellular uptake in cancer cells via endocytosis mediated by the interaction between HA and CD44 receptors.

Traditionally, the escape from endosomes/lysosomes has been considered a critical step for effective siRNA silencing due to the degradation of RNAase [23]. However, the pH‐responsive disassembly of AHA@MnP/siRNA nanoparticles could facilitate to release siRNA into the cytoplasm. CLSM images in Figure 2L illustrated the intracellular distribution, showing that the co‐localization of siRNA (Cy5‐labeled, red) and lysosomes (stained with LysoTracker, green) primarily occurred between 2 to 4 h. As time progressed, an increasing amount of siRNA was diffusely distributed in the cytoplasm by the 6 h time point. Based on these results, the acid‐responsive release and subsequent lysosomal escape mechanisms of nanoparticles were mapped (Figure 2M). After cellular uptake, the endosomes containing the internalized nanoparticles would fuse with early lysosomes. The acidic environment in lysosomes (pH 4.0–5.0) compared to endosomes (pH 6.0) could facilitate the disassembly of AHA@MnP/siRNA nanoparticles. This disassembly process would result in a significant increase in various ions, leading to an elevation in the inner osmotic pressure. Consequently, this would accelerate the release of siRNA and Mn^2+^ from late lysosomes into the cytoplasm. This observation validates our hypothesis that AHA@MnP/siRNA NPs can maintain stability under physiological conditions while disassembling into highly diffusive units in cancer cells, ultimately releasing the payload in a controlled manner within the endo/lysosomes.

There are serval studies revealed that the immune checkpoint of PD‐1/PD‐L1 inhibits the function of CD8^+^ T cells [18]. However, siPD‐L1 can downregulate the expression of PD‐L1 in cancer cells, reducing this inhibition. The gene silencing effect of AHA@MnP/siPD‐L1 nanoparticles in 4T1 cells was evaluated using Western blot analysis. The results showed that compared to the control group, AHA@MnP/siPD‐L1 nanoparticles effectively inhibited the expression of PD‐L1 by 85.30% (Figure 2N,O), which was significantly higher than that achieved by MnP/siPD‐L1 nanoparticles (44.51%, *p* < 0.01) and Lipo/siPD‐L1 (58.81%, *p* < 0.001). This superior efficacy was primarily attributed to the enhanced intracellular uptake and rapid lysosomal escape of AHA@MnP/siPD‐L1 NPs.

Above all, these advantages of AHA@MnP/siPD‐L1 NPs could reduce acidity, relieve hypoxia and unlock the immune inhibition of PD‐1/PD‐L1, which contributed to a combined “Brakes off” effect on reprogramming of the tumor microenvironment.

#### 2.2.2 The “Step on the Accelerator” Effect of AHA@MnP/siPD‐L1 NPs In Vitro

**FIGURE 3:**
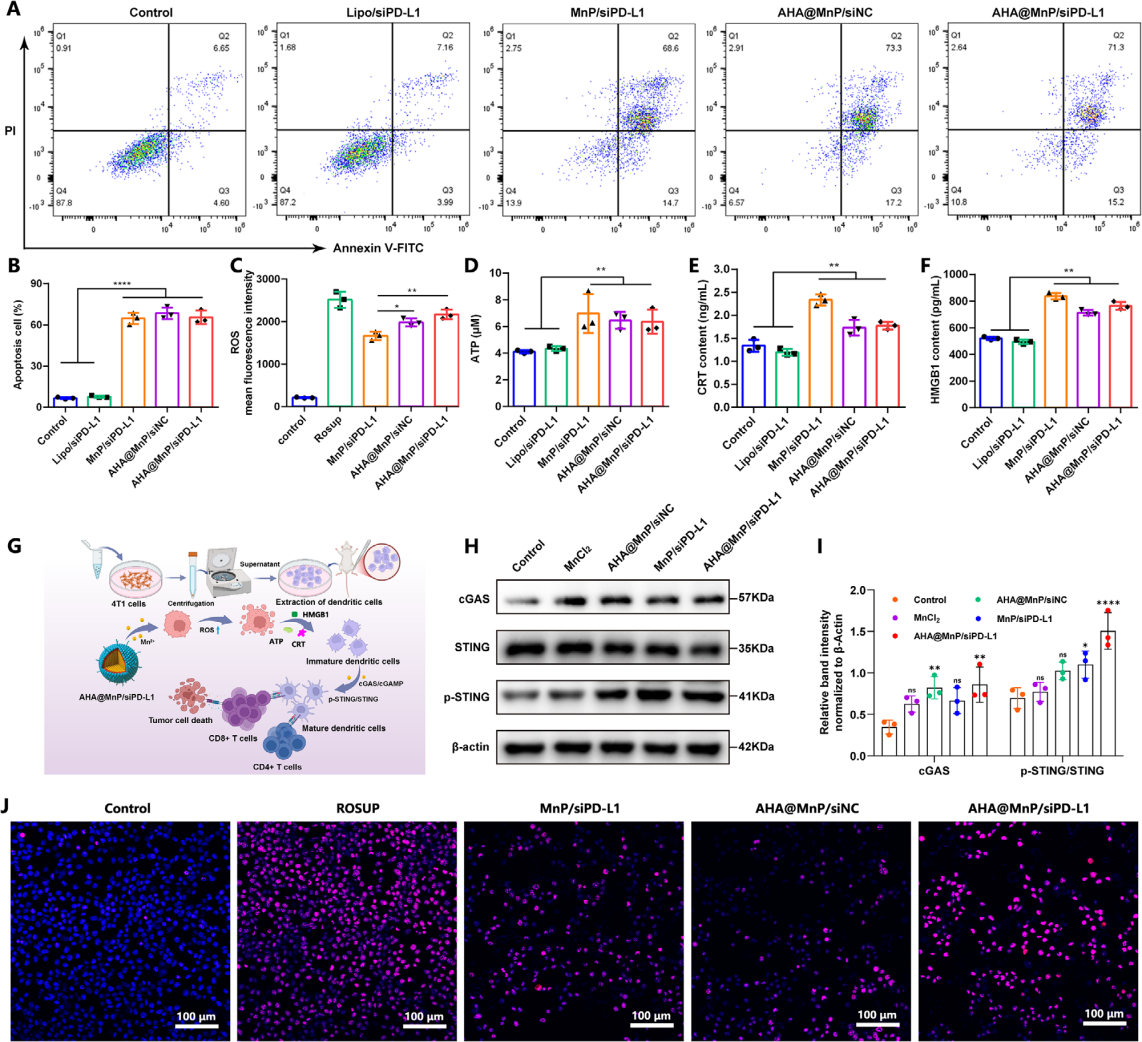
The “Step on the accelerator” effect of AHA@MnP/siPD‐L1 NPs in vitro immune therapy. (A,B) The apoptosis effect detected by flow cytometry analysis of Annexin V‐FITC/PI costained 4T1 cells (*n* = 3). The ROS level was detected by flow cytometry (C) and confocal microscopy (J) (red, ROS; blue, DAPI). (D) Quantification intracellular levels of ATP release, (E) levels of secreted CRT and (F) HMGB1 after treatment of various nanoparticles (*n* = 3). (G) Schematic diagram of cGAS‐STING pathway activation by AHA@MnP/siPD‐L1 NPs. (H) The expression of cGAS, p‐STING and STING protein for BMDC treated with AHA@MnP/siPD‐L1 NPs; (I) Quantitative analysis of the protein intensity rate of BMDC. Data are presented as the mean ± SD. *P* values were calculated using one‐way ANOVA. *p < 0.05, **p < 0.01, ***p < 0.001, and ****p < 0.0001.

To detect the “Step on the accelerator” effect of AHA@MnP/siPD‐L1 NPs in vitro, the therapeutic effects were analysed by the CCK‐8 cell counting kit, Annexin V‐FITC/PI flow cytometry and ICD apoptosis relative kits. Our results, illustrated in Figure S8A, showed significantly increased inhibition in 4T1 cells after 24 and 48 h of incubation (AHA@MnP/siPD‐L1 ≈ AHA@MnP/siNC < MnP/siPD‐L1 NPs), indicating the efficacy of Mn^2+^ in inhibiting tumor cells. Similarly, the survival rate of L929 cells remained above 80% after treatment, suggesting a favorable safety profile of the NPs (Figure S8B). The Annexin V‐FITC/PI results in Figure 3A–B further demonstrated the superior apoptosis‐inducing effect of Mn^2+^‐loaded NPs on 4T1 cells compared to Lipo/siPD‐L1 NPs (approximately three times). Overall, our findings support the effective in vitro cancer cell‐killing ability of AHA@MnP/siPD‐L1 NPs.

The mitochondria play a crucial role as the primary sites for generating reactive oxygen species (ROS), which are essential signaling molecules in biological systems for regulating oxidative stress homeostasis. In this study, the ROS levels in 4T1 cells were assessed following treatment with Mn^2+^‐loaded nanoparticles using flow cytometry and confocal laser scanning microscopy (CLSM). The results depicted in Figure 3C,J indicated a significant increase in ROS levels (≈10 times) in 4T1 cells treated with AHA@MnP/siPD‐L1 nanoparticles compared to the untreated group. This increase was comparable to that observed with AHA@MnP/siNC nanoparticles but slightly higher than with MnP/siPD‐L1 nanoparticles. Given the biological effects of ROS, it was hypothesized that Mn^2+^‐loaded nanoparticles could trigger immunogenic cell death (ICD) in 4T1 cells through mitochondrial damage leading to DNA damage or cyclic dinucleotides (CDN) formation.

To investigate the induction of ICD, several distinct biomarkers were evaluated using Elisa kits after incubating cells with different nanoparticles. As illustrated in Figure 3D–F, treatment with Mn^2+^‐loaded nanoparticles resulted in a significant upregulation of ATP, CRT, and HMGB1 levels in the cell culture medium, indicating the activation of ICD. Furthermore, previous studies have reported that Mn^2+^ could enhance the DNA recognition sensitivity of cyclic GMP‐AMP synthase (cGAS), leading to the activation of the cGAS‐STING pathway and maturation of the DCs [24]. Therefore, the mechanism should be that the released Mn^2+^ could disturb mitochondrial function and mediate the enhanced oxidative stresses, and then produce ROS to induce ICD in 4T1 cells, which will initiate immune responses sequentially, resulting in DCs maturation and the further activation and proliferation of CTLs (Figure 3G). To explore this ICD mechanism, we performed a series of protein blot analyses to assess key ICD biomarkers. As shown in Figure 3H–I, cGAS and p‐STING, and STING protein expression were significantly up‐regulated in bone marrow‐derived dendritic cells (BMDC) after different NPs treatments, suggesting activation of the ICD and the involvement of the cGAS‐STING signaling axis. This activation is essential for dendritic cell proliferation and maturation as well as T cell activation. Together, the AHA@MnP/siPD‐L1 NPs will mediate the “Step on the accelerator” effect on reprogramming the TME for a better anticancer treatment.

### 2.3 In Vivo Targeting and Biodistribution of AHA@MnP/siPD‐L1 NPs

**FIGURE 4:**
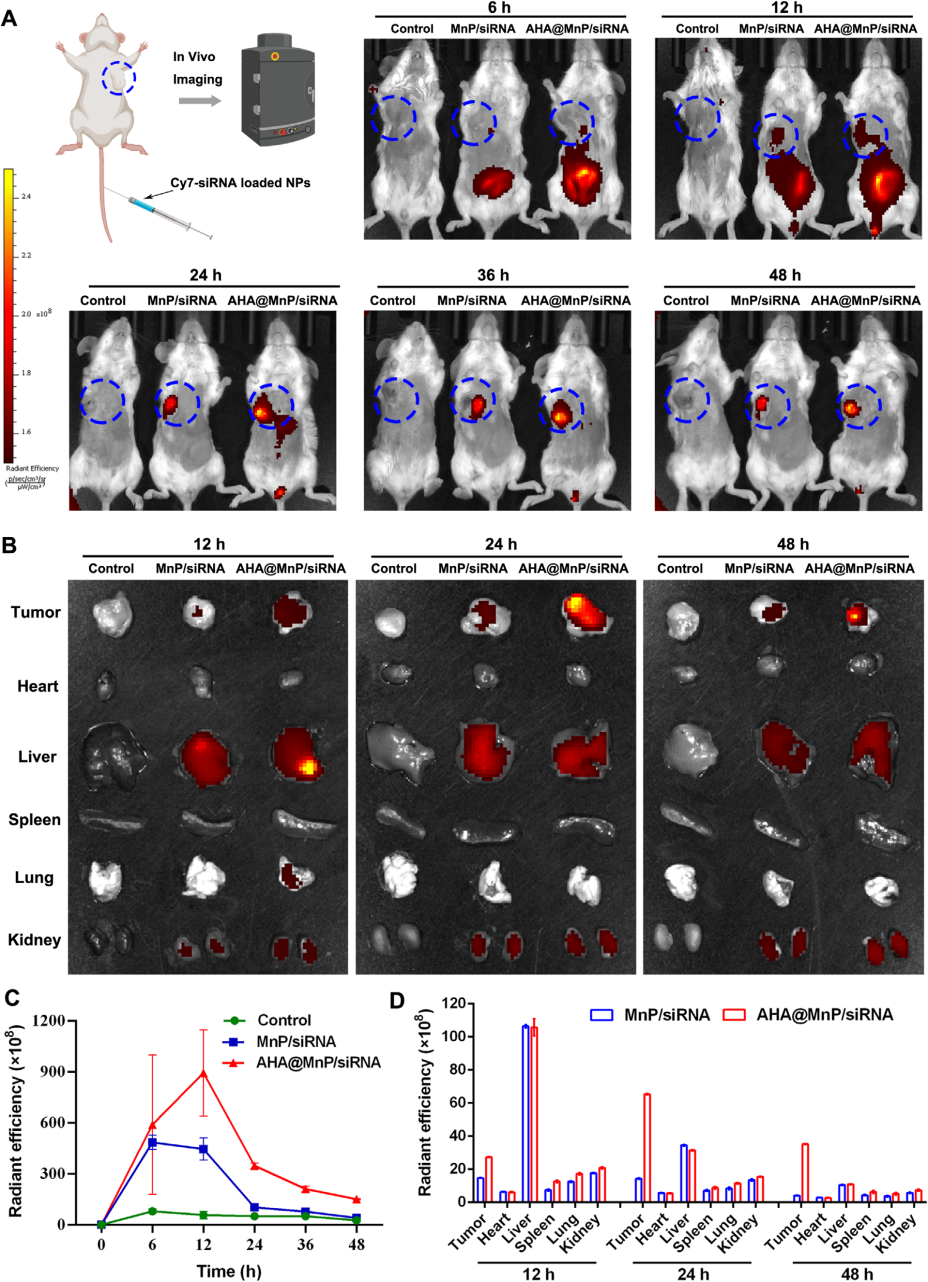
Determination of the targeting and biodistribution of AHA@MnP/siPD‐L1 NPs in vivo. (A) Representative fluorescence images of NP‐treated mice in vivo at different time points. (B) Fluorescence images of major organs and tumors at different time points. (C) Radiant efficiency of fluorescence at different time points. (D) Radiant efficiency of fluorescence in different organs. The mice bearing the 4T1 tumor were given intravenous injection via the tail vein. The dose of Cy7‐siRNA was 0.25 mg kg^−1^. Data are presented as the mean ± SD (*n* = 3).

Investigating the systemic biodistribution and clearance of nanotheranostic agents is essential for their potential biomedical applications. In this study, we aimed to assess the tumor‐targeting ability of AHA@MnP/siPD‐L1 NPs using Cy7‐labeled NPs (Cy7‐siPD‐L1) in 4T1 cancer cell‐bearing BALB/c mouse model. In vivo, fluorescence imaging with the IVIS spectrum system was conducted following the intravenous administration of Cy7‐labeled AHA@MnP/siPD‐L1 NPs or MnP/siPD‐L1 NPs (0.25 mg kg^−1^, equal fluorescent intensity) at predefined time points (6, 12, 24, 36, and 48 h). As shown in Figure 4A, the tumors exhibited a time‐dependent increase in Cy7 fluorescence signal, with higher intensity observed in mice treated with AHA@MnP/siPD‐L1 NPs compared to those treated with MnP/siPD‐L1 NPs, indicating enhanced targeting capability with AHA coating (Figure 4C). Following the injection of nanoparticles (NPs) for 12, 24, and 48 h, mice were sacrificed, and ex vivo fluorescence imaging of the primary organs (heart, liver, spleen, lung, and kidney) and the tumor was performed. The results revealed that the fluorescence intensity was notably higher in the tumor tissue at 24 and 48 h post‐injection, surpassing even the fluorescence intensity observed in the corresponding liver tissue. Moreover, the fluorescence intensity in isolated tumors treated with AHA@MnP/siPD‐L1 nanoparticles was significantly higher than that in tumors treated with MnP/siPD‐L1 nanoparticles (Figure 4B,D). Analysis suggested a progressive accumulation of AHA@MnP/siPD‐L1 NPs at the tumor site, likely attributed to two main factors: (1) prolonged circulation enhancing the EPR effect due to the electronegative AHA shell, and (2) the targeting capacity facilitated by the AHA‐CD44 membrane protein interaction in 4T1 cancer cells. These findings suggested that AHA@MnP/siPD‐L1 nanoparticles demonstrated favorable tumor‐targeting capabilities and pharmacokinetic profiles. The prolonged circulation in the bloodstream and enhanced efficiency in accumulating within tumor tissues highlighted their potential as promising nanotherapeutics for targeted cancer therapy.

### 2.4 In Vivo Anticancer Performance and Safety of AHA@MnP/siPD‐L1 NPs

**FIGURE 5:**
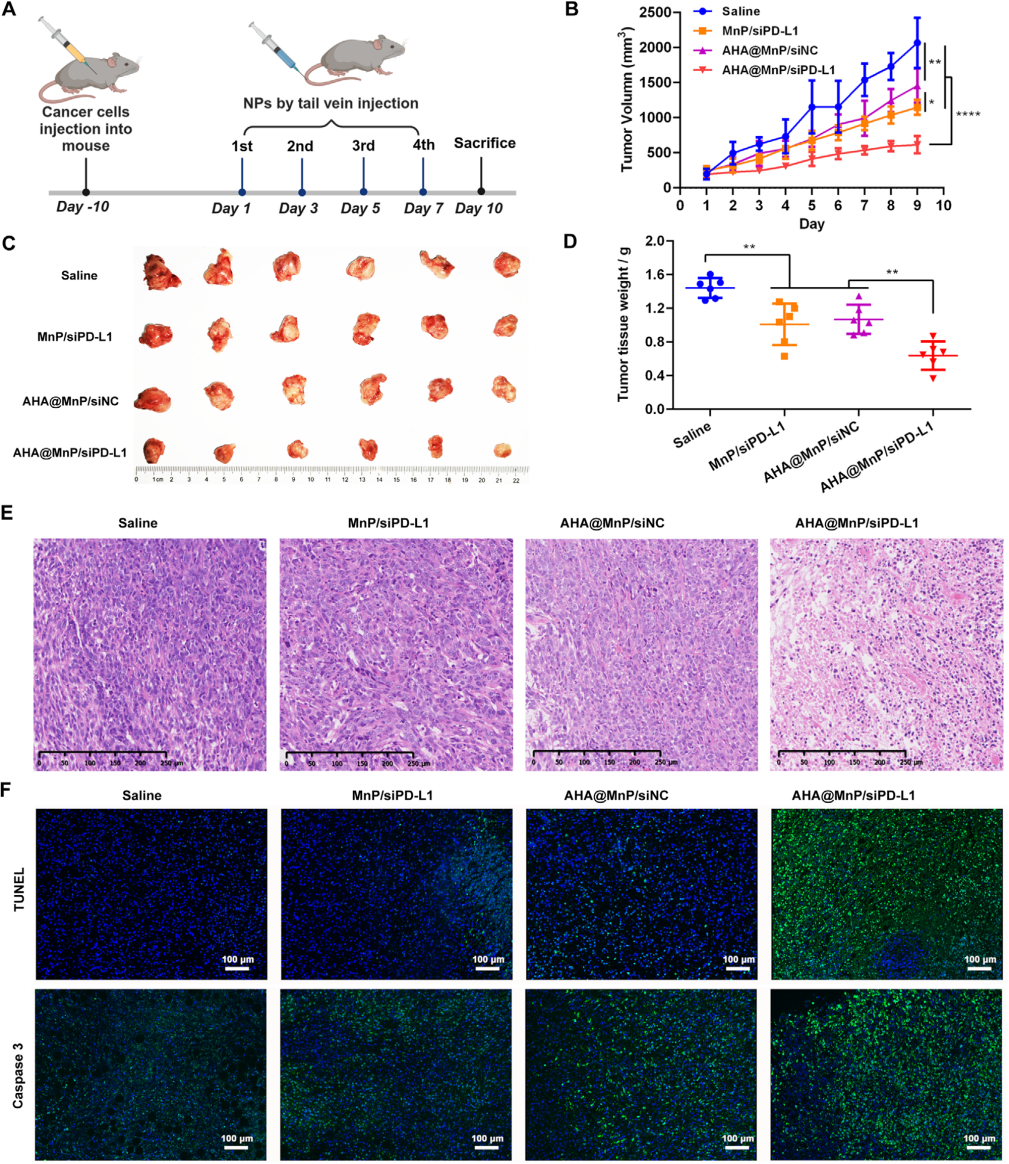
In vivo antitumor immunotherapy study of AHA@MnP/siPD‐L1 NPs in BALB/c mice bearing 4T1 cancer cells. (A) The treatment schedule for mice with different NPs. (B) Tumor growth curves after treated by various NPs (*n* = 6, siPD‐L1 or siNC: 0.25 mg kg^−1^). (C) Photographs of harvested tumor tissues (*n* = 6). (D) Tumor weight of harvested tumor tissues (*n* = 6). (E) Haematoxylin‐eosin staining of tumor. (F) Apoptosis levels (TUNEL and Caspase3 staining) (green, apoptotic cell; blue, DAPI) in tumor tissue. Data are presented as the mean ± SD. *P* values were calculated using one‐way ANOVA. *p < 0.05, **p < 0.01, ***p < 0.001, and ****p < 0.0001.

To evaluate the potency of AHA@MnP/siPD‐L1 NPs in vivo anti‐tumor therapy, 4T1 tumor‐bearing BALB/c mice were randomly allocated and injected relative NPs upon reaching a tumor volume of approximately 100 mm^3^ (*n* = 6): (1) Control (Saline); (2) MnP/siPD‐L1; (3) AHA@MnP/siNC; and (4) AHA@MnP/siPD‐L1 (siRNA: 0.25 mg kg^−1^) (Figure 5A). The therapeutic efficacy, as indicated by tumor size and body weight, was monitored daily. The MnP/siPD‐L1, AHA@MnP/siNC and AHA@MnP/siPD‐L1 groups inhibited varying degrees of tumor inhibition compared to the saline group (Figure 5B). However, AHA@MnP/siPD‐L1 NPs exhibited the best antitumor effects. Compared to the control group, the tumor growth inhibition rate in MnP/siPD‐L1, AHA@MnP/siNC and AHA@MnP/siPD‐L1 groups was 44.50%, 29.62% and 70.34%, respectively. At the end of the treatment period, the mice were euthanized, and tumor samples were collected for further analysis (Figure 5C–D), confirming the robust antitumor efficacy of AHA@MnP/siPD‐L1 NPs and the other two NPs. The observed suboptimal tumor elimination or regression in some cases may be attributed to the relatively lower doses of siPD‐L1 and Mn^2+^ administered compared to prior studies [11, 25]. Furthermore, the H&E staining of tumor tissue post‐treatment indicated that the cellular arrangement was most relaxed in the AHA@MnP/siPD‐L1 NPs group, exhibiting notably reduced nuclear staining and increased nuclear fragmentation compared to the other treatment groups (Figure 5E). Moreover, the assessment of tumor apoptosis was conducted using terminal‐deoxynucleotidyl transferase‐mediated nick end labeling (TUNEL) and caspase‐3 activity analysis. The results presented in Figure 5F demonstrated a noticeable induction of apoptosis following treatment with the three Mn^2+^‐loaded NPs. Particularly, AHA@MnP/siPD‐L1 NPs exhibited significantly higher rates of apoptosis compared to MnP/siPD‐L1 and AHA@MnP/siNC NPs. These results suggested that the extensive cancer cell death induced by AHA@MnP/siPD‐L1 NPs resulted from the synergistic effects of Mn^2+^ and siPD‐L1.

The evaluation of treatment toxicity and biosafety is crucial in preclinical studies. Throughout the treatment period, the body weights of mice in all four groups were relatively stable, as depicted in Figure S9A. Additionally, systematic assessments of biosafety, including blood routine, blood chemistry, and histological examination of main organs, were conducted. The results presented in Figures S9B to S9L demonstrated that blood biochemistry indicators were similar across the four groups. Furthermore, H&E staining of the main organs revealed no apparent damage or lesions in mice from any of the treatment groups, as illustrated in Figure S10. In conclusion, AHA@MnP/siPD‐L1 NPs exhibited potent antitumor effects with favorable biosafety profiles.

### 2.5 In Vivo Immunotherapeutic Anticancer Performance of AHA@MnP/siPD‐L1 NPs

**FIGURE 6:**
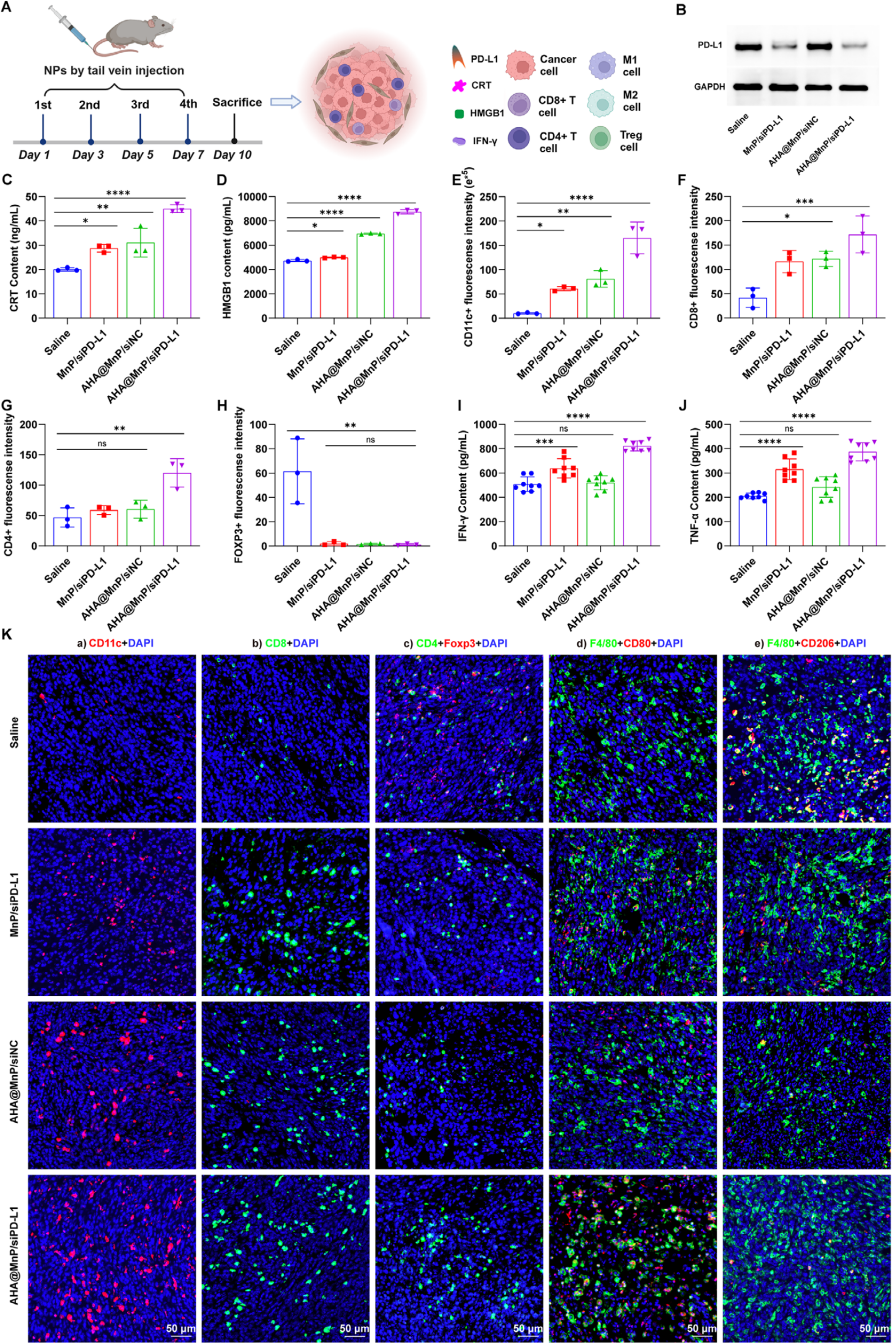
The synthetical effect of AHA@MnP/siPD‐L1 NPs in vivo immune therapy. (A) The treatment schedule for mice and tumor tissue. (B) The expression of the PD‐L1 protein in tumor detected by the western‐blot assay (*n* = 3). (C) Levels of CRT and (D) HMGB1 in tumor (*n* = 3). Fluorescence intensity of (E) DCs cells (CD11c, red), (F) CD8^+^ T cells (CD8, green) and (G) CD4^+^ T cells (CD4, green) in tumor (*n* = 3). (H) Fluorescence intensity of Treg Cells (FOXP3, red) in CD4^+^ cells (*n* = 3). (I) Levels of IFN‐γ and (J) TNF‐α in serum (*n* = 8). (K) Fluorescence imaging of various immune cell subpopulations. that is, CD11c^+^: Dendritic cell, CD8^+^: CD8 T cells, CD4^+^: CD4 T cells, Foxp3^+^: Treg Cells, CD80^+^: M1 macrophages cells, CD206^+^: M2 macrophages cells, cell nucleus was stained with DAPI (blue). Data are presented as the mean ± SD. *P* values were calculated using one‐way ANOVA. *p < 0.05, **p < 0.01, ***p < 0.001, and ****p < 0.0001.

The bidirectional reprogramming of the tumor microenvironment (TME) by AHA@MnP/siPD‐L1 NPs was further investigated by extracting whole proteins from tumor tissues obtained from the experimental groups. The evaluation of ICD was conducted, and various immune cell subpopulations were identified and analyzed using fluorescence staining based on canonical markers of immune cell functions, as depicted in Figure 6A. Consistent with the in vitro results, the PD‐L1 knockdown rates in the MnP/siPD‐L1, AHA@MnP/siNC, and AHA@MnP/siPD‐L1 groups were 56.09%, −10.59%, and 71.11%, respectively, compared to the control group (saline), as shown in Figure 6B and Figure S11. Furthermore, Mn^2+^‐loaded NPs could significantly up‐regulate the levels of CRT and HMGB1, indicating the activation of ICD (Figure 6C–D). The release of Mn^2+^ was more favorable for cGAS‐STING pathway activation and DCs maturation which further promoted the initiation and activation of T cells, and the inhibition of suppressive immune cells [8a]. Consequently, the infiltration levels of antitumor‐related immune cells, including helper CD4^+^ T cells, killer CD8^+^ T cells, regulatory T cells (Tregs) and others, were further assessed using fluorescence staining to provide a comprehensive understanding of the immune cell landscape within the TME following treatment with AHA@MnP/siPD‐L1 NPs. The fluorescence intensity value of CD11c^+^ (a marker of DCs) was increased 16.5‐fold in AHA@MnP/siPD‐L1 NPs compared to the control group (Figure 6E), suggesting that it could promote the maturation of DCs. The percentage of CD8^+^ and CD4^+^ cells serves as a crucial marker for the initiation and activation of T cells. The analysis provided valuable information on the impact of AHA@MnP/siPD‐L1 NPs on T cell initiation and activation, further elucidating the immunomodulatory effects of the treatment in the context of antitumor immune responses. The fluorescence intensity of CD8^+^ T cells was increased in MnP/siPD‐L1 (282%) and AHA@MnP/siNC (291%) compared to the control group (fluorescence intensity of 100%), reaching the highest in the AHA@MnP/siPD‐L1 group (420%) (Figure 6F,K(a)) indicating that AHA@MnP/siPD‐L1 NPs could effectively increase the infiltration of CD8^+^ T cells. The percentage growth of CD4^+^ T cells was analogous to that of CD8^+^ T cells (Figure 6G,K(b)). However, the fluorescence intensity of immunosuppressive Treg (characterized by Foxp3^+^ and CD4^+^) in MnP/siPD‐L1, AHA@MnP/siNC, and AHA@MnP/siPD‐L1 NPs was significantly lower than that of the control group (61.5) at 1.95, 1.67, and 1.10, respectively (Figure 6H,K(c)). Furthermore, the percentage of M1 macrophages (CD80 marker, positive immune cells, Figure 6K(d)) increased but M2 macrophage (CD206 marker, immunosuppressive immune cells, Figure 6K(e)) decreased remarkedly in three Mn^2+^ loaded NPs groups (especially in AHA@MnP/siPD‐L1 NPs), probably because that the increase of ROS in tumor tissue might contribute to the polarization of M2 macrophages towards an M1 phenotype. This shift in macrophage polarization could further enhance the antitumor immune response by promoting a pro‐inflammatory environment. Additionally, serum samples obtained from the various treatment groups were analyzed for cytokine content using enzyme‐linked immunosorbent assay (ELISA). The significantly elevated levels of tumor necrosis interferon‐γ (IFN‐γ) and factor‐α (TNF‐α) observed in the AHA@MnP/siPD‐L1 NP‐treated group indicated the potent enhancement of T cells infiltration and function (Figure 6I–J). These findings suggested that AHA@MnP/siPD‐L1 NPs effectively stimulated a robust immune response, further supporting their immunomodulatory properties in the context of antitumor immunity.

### 2.6 In Vivo Immunotherapeutic Anticancer Molecular Mechanism of AHA@MnP/siPD‐L1 NPs

In order to further explore the molecular mechanism of its anti‐tumor immunity, bulk RNAseq analysis was performed on cells isolated from tumors after treatment with different groups (P group: AHA@MnP/siPD‐L1 NPs, S group: Saline, N group: AHA@MnP/siNC NPs).

**FIGURE 7:**
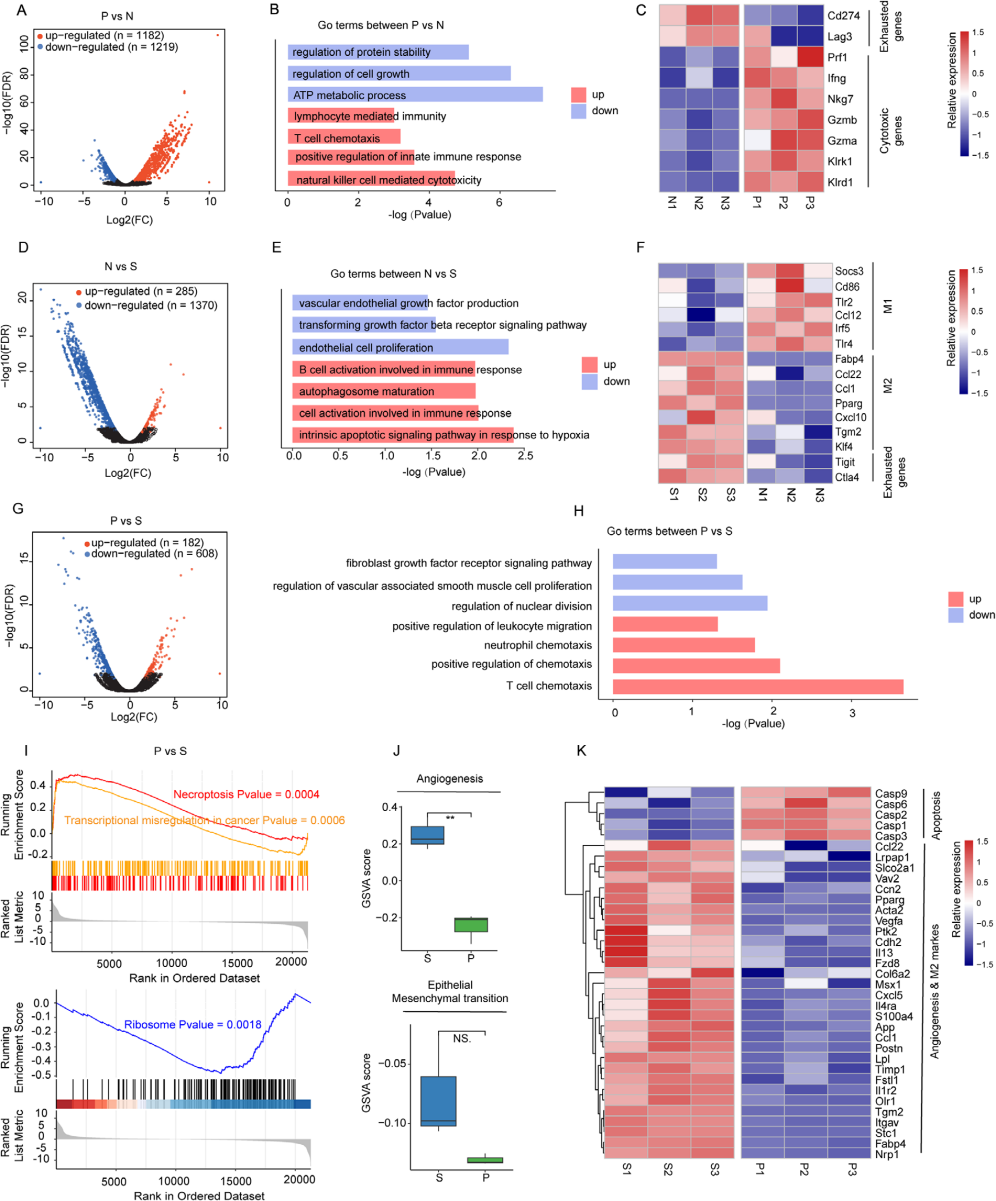
The synthetical effect of AHA@MnP/siPD‐L1 NPs in vivo immune therapy. (A) The volcano plots show the DEGs between P versus N. The *x*‐axis illustrates the log2 (fold change (FC)), and the *y*‐axis indicates as −log10 (*p‐*value). (B) The bar plots depicting enriched Go terms of upregulated and downregulated genes between P versus N. (red, up; blue, down). (C) Heatmap depicts T cell cytotoxic genes and exhausted genes expression levels across N and P groups. (D) The volcano plots show the DEGs between N versus S. The *x*‐axis illustrates the log2 (fold change (FC)), and the *y*‐axis indicates as −log10 (*p* value). (E) The bar plots depicting enriched Go terms of upregulated and downregulated genes between N versus S. (red, up; blue, down). (F) Heatmap depicts T cell exhausted genes, M1 and M2 related genes expression levels across N and S groups. (G) The volcano plots show the DEGs between P versus S. The *x*‐axis illustrates the log2 (fold change [FC]), and the *y*‐axis indicates as −log10 (*p* value). (H) The bar plots depict enriched Go terms of upregulated and downregulated genes between P versus S (red, up; blue, down). (I) The GSEA plot demonstrates that P group up‐regulated Necroptosis and Transcriptional mis‐regulation in the cancer signaling pathway and down‐regulated ribosome signaling pathway compared with S group. (J) The plots show the distribution of enrichment scores of Angiogenesis and Epithelial mesenchymal transition between the S (blue) and the P (green) groups. Data are shown as mean ± SD. (K) Heatmap depicts Apoptosis related genes, M2 and Angiogenesis related genes expression levels across P and S groups. S group: Saline, P group: AHA@MnP/siPD‐L1, N group: AHA@MnP/siNC.

First, we investigated the differentially expressed gene (DEG) analysis between P and N group, and revealed a total of 2461 (1182 up and 1219 down) DEGs (Figure 7A). Gene ontology (Go) enrichment of these upregulated genes in P group revealed activated pathways that are associated with immune cell activation, such as lymphocyte mediated immunity, T cell chemotaxis and natural killer cell mediated cytotoxicity (Figure 7B). Next, the exhausted and cytotoxic associated expression patterns of these samples in P and N groups were examined [26] (Figure 7C). Compared with the N groups, cytotoxic genes (Klrd1, Klrk1 and so on) exhibited higher expression in the P groups. On the contrary, genes related to T cell exhausted were more expressed in the N groups. These changes could be attributed to the silencing of PD‐L1 expression by siPD‐L1, breaking the immunosuppressive effect of PD‐1/PD‐L1 to release the activity of CD8+ T cells.

Moreover, to understand the effect of Mn^2+^ on tumor cells, the DEG analysis between N and S groups was conducted (Figure 7D). Pathway enrichment revealed that intrinsic apoptotic signaling pathway in response to hypoxia was significantly up‐regulated in the N group, while endothelial cell proliferation and transforming growth factor beta (TGF‐β) receptor signaling pathway were down‐regulated (Figure 7E). TGF‐β receptor signaling pathway had been reported in the literature to be associated with the epithelial‐mesenchymal transitions (EMTs) process during tumor development [27], implying that Mn^2+^ could inhibit tumor cell proliferation and the EMT process. Heatmap depicts T cell exhausted genes, M1 and M2 related genes expression levels across N and S groups [28]. the results showed the elevated expression levels of M1‐related genes up‐regulated, while M2‐associated genes down‐regulated after treatment of the N group (Figure 7F), indicating that Mn^2+^ could increase expression of M1‐related genes and inhibit the expression of M2‐related genes and TGF‐β pathway for better anti‐tumor therapy.

Finally, to observe the impact of the molecule on tumor cells, we performed DEGs analysis between P and S groups (Figure 7G). Go enrichment analysis was performed based on the corresponding DEGs to further investigate the alterations in biological processes in mice with 4T1 cancer and those treated with P (Figure 7H). We found that several immune‐associated pathways such as T cell chemotaxis, neutrophil chemotaxis and positive regulation of leukocyte migration were up‐regulated, while pathways related to cancer progression such as regulation of nuclear division and regulation of vascular‐associated smooth muscle cell proliferation were down‐regulated after treatment of P groups. Then, the gene set enrichment analysis (GSEA) based on DEG of P versus S showed that necroptosis and transcriptional misregulation in cancer were upregulated in the P group (*p* value < 0.05), and ribosome was downregulated in the P group (*p* value < 0.05). These findings imply that P has the function of inducing tumor cell apoptosis (Figure 7I). Next, the Gene Set Variation Analysis (GSVA) score of angiogenesis and epithelial‐mesenchymal transition in the bulk‐RNA dataset suggested that the P group displayed lower scores than the S group (Figure 7J). The expression levels of representative genes indicative of Angiogenesis & M2 markers and Apoptosis manifested that Angiogenesis and M2 markers were downregulated and Apoptosis related genes were upregulated in the P group (Figure 7K). These results indicated that the P group could inhibit tumor cell angiogenesis and M2 characteristics to prevent the development of cancer.

Collectively, these results indicated that the AHA@MnP/siPD‐L1 NPs could obtain the synergistic immunotherapeutic effect mainly based on the TME bidirectional reprogramming capacity: (1) overloaded Mn^2+^ induce apoptosis, enhance the activity of M1 and promote the proliferation and activation of CD8^+^ T cells, but inhibit the activity of M2 and TGF‐β pathway; (2) siPD‐L1 knockdown the PD‐L1 expression of tumor cells to restore the function of immune cells (CD8^+^ T cells and NK cells).

## 3 Conclusion

In summary, the pH‐responsive core‐shell manganese phosphate nanomodulator, AHA@MnP/siPD‐L1 NPs, was designed to enhance anti‐tumor immunotherapy through bidirectional reprogramming of the TME (Scheme 1). First, these NPs effectively scavenged protons to counteract tumor acidity, thereby elevating the pH of the TME. Second, the release of Mn^2+^ from the NPs led to the consumption of hydrogen peroxide (H_2_O_2_) and the generation of ROS, triggering ICD and activating the immune response. Meanwhile, Mn^2+^ was more favorable for cGAS‐STING pathway activation and DCs maturation which further promoted the initiation and activation of CD8^+^ T cells, CD4^+^ T cells and M1 macrophage, and the inhibition of suppressive immune cells (M2 macrophage and Tregs). Third, siPD‐L1 could knockdown the PD‐L1 expression to unlock the immune inhibition of PD‐1/PD‐L1 for a better cancer‐killing effect. Finally, based on the target capability of HA with CD44 over‐expressed in cancer cells, the AHA@MnP/siPD‐L1 NPs exhibited significant accumulation at tumor sites with good biosafety and demonstrated potent antitumor effects against 4T1 tumors. Nevertheless, it is notable to check the anti‐tumor therapeutic effect in more tumor models with higher doses of prepared NPs in subsequent studies. Above all, the bidirectional reprogramming TME (“Brakes off”, restoring CD8^+^ T cells function and “Step on the accelerator”, promoting CD8^+^ T cells infiltration) increases the combined immunotherapy, suggesting that the manganese phosphate nanomodulator (AHA@MnP/siPD‐L1 NPs) was an effective and potential strategy to enhance antitumor immune response in poorly immunogenic tumors, and provided the multimodal clinical solid tumors treatment.

## 4 Methods

All methods are available in Supporting Information.

## Author Contributions

C.Q., F.X., Y.L., and Q.T. designed the projects and performed experiments, collected and analyzed data, and wrote the manuscript. C.W., S.L., H.Y., L.Z., Y.F., P.G., Y.H., X.Z., L.Z., L.G., W.C., J.Y., J.Z., H.P, Y.M., Q.S. and C.P. assisted in experiments and provided some helpful suggestions. C.Q., J.W., X.T. and Z.G. reviewed the manuscript. The manuscript has been read and approved by all co‐authors.

## Ethics Statement

All animal studies were approved by the Institutional Animal Care and Use Committee and Animal Ethics Committee of the Institute of Chinese Materia Medica (Approve number 2024B221).

## Conflicts of Interest

The authors declare no conflicts of interest.

## Supporting information



Supporting Information

## Data Availability

The data that support the findings of this study are available from the corresponding author upon reasonable request.
