# Supplementary material for: Cancer Immunotherapy Based on the Bidirectional Reprogramming of the Tumor Microenvironment by a “Brakes Off/ Step on the Accelerator” Core‐Shell Manganese Phosphate/siPD‐L1 Modulator
Source: Exploration (Beijing). 2025 Feb 9;5(3):270009. doi: 10.1002/EXP.70009 (PMC12199384; doi:10.1002/EXP.70009)
Supplement: Supplementary file 1 — Supporting Information [file EXP2-5-270009-s001.docx]

**Supporting Information**

**Cancer immunotherapy based on** **the bidirectional reprogramming of the tumor microenvironment by a “Brakes off/ Step on the accelerator” core-shell** **manganese phosphate/siPD-L1 modulator**

*Fei Xia^#1^, Yuqian Lu^#1^*, *Zipeng Gong^#2^, Qingchao Tu^#1,3^, Shuntao Liang^4^, Chen Wang^1^, HaiLu Yao^1^, LinYing Zhong^1^, Yuanfeng Fu^1^, Pengbo Guo^1^, Yichong Hou^1^, Xinyu Zhou^1^, Li Zou^1^, Licheng Gan^1^, Weiqi Chen^1^, Jiawei Yan^1^, Junzhe Zhang^1^, Huanhuan Pang^1^, Yuqing Meng^1^, Qiaoli Shi^1^, Chen Pan^1^, Xiaomei Tao^*4^, Jigang Wang^*1,3,5^, Qingfeng Du^*6^,Chong Qiu ^*1^*

*^1^ State Key Laboratory for Quality Ensurance and Sustainable Use of Dao-di Herbs, Artemisinin Research Center, and Institute of Chinese Materia Medica, China Academy of Chinese Medical Sciences, Beijing 100700, China.*

*^2^ State Key Laboratory of Discovery and Utilization of Functional Components in Traditional Chinese Medicine, Guizhou Provincial Engineering Research Center for the Development and Application of Ethnic Medicine and Traditional Chinese Medicine, Guizhou Medical University, Guiyang 561113, China*

*^3^ State Key Laboratory of Antiviral Drugs, School of Pharmacy, Henan University, Kaifeng 475004, China*

*^4^ BeiJing Shijitan Hospitals, Capital Medical University, Beijing 100038, China.*

*^5^* *Department of Nephrology, Guangdong Provincial Clinical Research Center for Geriatrics, Shenzhen Clinical Research Center for Geriatric, Shenzhen People’s Hospital (The Second Clinical Medical College, Jinan University; The First Affiliated Hospital, Southern University of Science and Technology), Shenzhen, Guangdong 518020, China.*

*^6^ Department of Traditional Chinese Medicine and School of Pharmaceutical Sciences, Southern Medical University, Guangzhou, Guangdong 510515, China.*

**Materials and Reagents**

1-Ethyl-3-(3-dimethyllaminopropyl)-carbodiimide (EDC) was obtained from J&K Scientific Ltd. (Beijing, China). Hyaluronic acid (HA, MW=780 kDa) was purchased from HuaXiFuRuiDa Ltd. (Shandong, China). Phosphate buffer saline (PBS) and Opti-MEM were purchased from Macgene (Beijing, China). Agarose was obtained from GENE COMPANY (Hong Kong, China); Hoechst 33258, Hoechst33342 and Lyso-tracker green were purchased from Molecular Probes Inc. (Oregon, USA); Rhodamine phalloidin was purchased from Invitrogen (Carlsbad, USA). Anti-PD-L1 siRNA (sense strand: 5′-GAGGUAAUCUGGACAAACATT-3′ and antisense strand: 5′-UGUUUGUCCAGAUUACCUCTT-3′, named as siPD-L1), negative control siRNA (sense strand: 5′-UUCUCCGAACGUGUCACGUTT-3′; antisense strand: 5′-ACGUGACACGUU CGGAGAATT-3′, named as siNC) and fluorescein-labeled siRNA (5′ end of the sense strand, FAM-siRNA or Cy5-siRNA) were synthesized and purified with HPLC by Gene Pharma Co. Ltd (Shanghai, China). Cell culture reagents and materials were purchased from M&C GENE TECHNOLOGY (Beijing, China).

**Cell lines**

Mice breast cancer 4T1 cells were provided by the Institute of Basic Medical Science, Chinese Academy of Medical Sciences (Beijing, China). The cells were cultured in the standard cell medium recommended by American Type Culture Collection (ATCC), at 37 ºC in a 5% CO_2_ atmosphere.

**Synthesis of alendronate-hyaluronan graft polymers (AHA)**

The alendronate-hyaluronan graft polymers (AHA) with the molecular weight of hyaluronan (HA_780k_) were synthesized. Briefly, HA (2 g, 2.5 μmol) was dissolved in 100 mL of degassed deionized water. EDC (581 mg, 3.75 mmol) and NHS (431 mg, 3.75 mmol) were slowly added in the HA solution. After 20 min stirring, alendronate sodium (1.36 g, 5.0 mmol) was added in the mixture. The reaction solution was adjusted at pH 4~5 and kept stirring for 2 days at room temperature. After the end of reaction, the product was purified by dialysis (MWCO = 8000 Da) against distilled water and lyophilized，obtained 1.8g of the final product with a 54% yield of alendronate. The structures of all products were confirmed by ^1^H-NMR (AVANCE III, 500 MHz, Bruker, Billerica, MA) spectrometry.

**Preparation and Optimization of** **AHA@MnP/siRNA NPs**

Briefly, 20 μL of MnCl_2_ solution (100 mM, pH 7.4) and 20 μL of siRNA solution (24 μM, diluted in water) were mixed, and then 80 μL of HEPES buffered solution (HBS) (50 mM HEPES, 280 mM NaCl, 1.5 mM Na_2_HPO_4_, pH 7.4) containing the predetermined amount of AHA was subsequently added under a one minute’s ultrasonic mixing at room temperature. For the preparation of MnP/siRNA coprecipitation control, the identical procedure was conducted except that no AHA mixture was added in HEPES buffer solution to stabilize the particles.

For optimizing the formulation of AHA@MnP/siRNA NPs, the size distribution with different concentration of AHA and Mn^2+^ were detected by a Zetasizer Nano ZS (Malvern, U.K.). Moreover, the siRNA-loading capability at different concentration of Mn^2+^ was detected by the Quant-iT™ RiboGreen® RNA Reagent and Kit.

**Characterization of AHA@MnP/siRNA NPs**

The size distribution of the various NPs was detected by a Zetasizer Nano ZS (Malvern, U.K.). The transmission electron microscope (TEM, JEM1400PLUS, Japan) and the scanning electron microscope (SEM, NovaNanoSEM430, USA) were used to observe particle size and morphology of the NPs. Then, the chemical components were characterised by XPS. The integrative hybrid nanostructure of AHA@Mn/siRNA NPs was further determined by UV-vis spectra and FT-IR analysis.

Additionally, to investigate the protective effects of different NPs on siRNA, the gel retardation assay was conducted. The AHA@Mn/siRNA NPs (siRNA: 2.4 μM) were mixed with fetal bovine serum at the volume ratio of 1: 1 and incubated for different times at 37 °C. The sample (20 μL) was sucked out at different time (1, 2, 3, 6, 9, 12, 24, 36 and 48 h) and kept in -20 °C before the gel retardation assay. Briefly, the samples (20 μL) were tackled with 4 μL 6×loading buffer or loading buffer containing 0.1 M HCl and electrophoresed on a 1 % agarose gel containing 0.5 mg mL^-1^ Exred (a special luminant dye for siRNA staining). Electrophoresis was performed at 80 V for 3 min, subsequently 100 V for 10 min, and these resulting gels were photographed under UV-illumination. Free siRNA (21 bp) was used as the control.

**Physical stability of AHA@MnP/siRNA NPs**

The physical stability of AHA@MnP/siRNA NPs (Mn^2+^:100 mM) was investigated as followed. Firstly, for the stabilities in different media, the NPs were diluted at the ratio of 1:9(v/v) with opti-MEM, mixture of PBS (K_2_HPO_4_ 1.06 mM, NaCl 155 mM, Na_2_HPO_4_ 3 mM, pH 7.4) and FBS (1:1, v/v), DMEM medium containing 10% FBS. After incubated at 37 ^o^C for 6 h, the particle size was monitored by dynamic light scattering (DLS). Secondly, for the stability of dilution, the NPs were diluted with opti-MEM medium at a series of ratios (1:7, 1:15, 1:31, 1:63, 1:127, 1:255 and 1:511(v/v)) and incubated at 37 ^o^C for 6 h, and then the particle size was monitored by DLS. Thirdly, for long-term stability of storage, the NPs suspensions was stored in Opti-MEM medium at room temperature, and record the change of size at predetermined intervals (1, 2, 3, 4, 5, 6 and 7 days) by DLS analysis.

**In Vitro Drug Release**

The releases of siRNA and Mn^2+^ from AHA@MnP/siRNA nanoparticles were investigated. The AHA@MnP/siRNA NPs were suspended in the release media with different pH (5.0, 5.5, 6.8 and 7.4) HEPES buffered solution (HBS). After incubation for a predetermined time period (0.5, 1, 2, 3, 4, 6, 8, 12, 24, 48 h), the samples were collected and centrifuged at 12000 g for 30 min, and the released siRNA in supernatant was analyzed by the Quant-iT™ RiboGreen® RNA Reagent and Kit; the concentration of Mn^2+^ in the supernatant was measured spectrophotometrically using potassium periodate.

**The pH-dependent disassembling of AHA@MnP/siRNA NPs**

The AHA@MnP/siRNA NPs were diluted in Opti-MEM medium with a series of pH values (5.0, 5.5, 6.0, 6.3, 6.5, 6.8, 7.0 and 7.4) at the ratio of 1:7 (v/v) and incubated for 15 min at room temperature, and then the particle size was monitored by DLS. Meanwhile, the above-mentioned samples treated with different pH values (7.4, 6.8, 5.5, 5.0) were drooped on a copper grid and allowed to dry under vacuum overnight, then a JOEL 100CX transmission electron microscope (100 kV) was used to image the samples.

**Cellular Uptake assay**

Flow cytometry was used to assess the quantitative cellular uptake. 4T1 cells were seeded 2.5×10^5^ per well in 12-well plates. After 24 h proliferation, the NPs containing FAM-labeled siRNA at a final concentration of 100 nM were exposed to cells and incubated for an additional appointed time at 37 ^o^C. After incubation, the cells were harvested and washed three times with pre-cooled PBS solution, and intracellular fluorescence intensities were detected by a FACS Calibur flow cytometry (Becton Dickinson, San Jose, CA, USA) immediately. Meanwhile, the intracellular fluorescence distribution was visualized under a Leica TCS SP8 confocal fluorescence microscope (Leica Microsystems, Heidelberg, Germany).

**Lysosomal escape assay**

4T1 cells (2.5×10^5^ cells per well) were seeded into confocal dishes. After 24 h incubation, the NPs of Cy5-labeled siRNA (200 nM) in Opti-MEM were added into each dish for different time periods (2, 4, 6, 8 h). At the end of transfection, LysoTracker Red (Invitrogen,Carlsbad, USA) (250 nM) was added in cell culture medium and incubated for 15 min at 37 ^o^C for endo/lysosome labeling. Next, the cells were washed with PBS for three times. Intracellular distribution of Cy5-labeled siRNA was observed with a Leica SP8 confocal microscope (Leica Microsystems, Heidelberg, Germany).

**In Vitro Cytotoxicity Study**

The cytotoxicity of NPs was determined via CCK-8 assay. Briefly, 4T1 cells were seeded in 96-well plates at a density of 8000 cells per well for 24 h proliferation. The cells were treated with 100 μL Opti-MEM containing various NPs (the final concentration of siRNA 100 nM) for 2 hours incubation. After that, the medium was replaced by DMEM medium containing 10 % FBS and the cells were continuously incubated for 24 or 48 h. Cell viability was evaluated by a CCK-8 kit (Solarbio Science & Technology Co. Ltd., Beijing, China) according to the manufacturer’s protocol.

**Acidic Attenuation Effect of NPs in vitro**

The as-prepared AHA@MnP/siRNA NPs was suspended in HEPES buffer solution with different pH values (pH 5.4, 6.5 and 7.4), stirring at RT and evaluating the pH value using a pH meter at default timepoints (0, 0.5, 1, 2, 6, 12, 24 and 48 h).

**Quenching of H_2_O_2_ by NPs**

For the quenching experiment, MnP/siRNA NPs and AHA@MnP/siRNA NPs (50 μM Mn^2+^) was suspended in PBS, H_2_O_2_ (100 μM) was added to initiate the reaction. For in vitro reactivity of as-prepared NPs toward H_2_O_2_, the concentration of H_2_O_2_ was measured using Hydrogen Peroxide Assay Kit (Beyotime Biotechnology, China). For in vitro cellular reactivity of as-prepared NPs toward H_2_O_2_, 4T1 cells (5.0 × 10^5^ cells per well) were seeded into 6-well tissue culture plates. After 24 h proliferation, various NPs containing siPD-L1 (100 nM) were exposed to cells and incubated for an additional 2 h at 37 °C. After refreshed cell culture medium, the cells were incubated for another 12 h of proliferation. The amount of intracellular H_2_O_2_ was quantified using Hydrogen Peroxide Assay Kit.

**Detection of ROS Generation**

4T1 cells (5.0 × 10^5^ cells per well) were seeded into 6-well tissue culture plates. After 24 h proliferation, various NPs containing siPD-L1 (100 nM) were exposed to cells and incubated for an additional 2 h at 37 ^o^C, and cells were incubated with DCFH-DA (Beyotime Biotechnology, China) for 30 min at 37 °C. ROS generation was determined by flow cytometry and confocal microscopy.

**Detection of ATP levels**

4T1 cells (5.0 × 10^5^ cells per well) were seeded into 6-well tissue culture plates. After 24 h proliferation, various NPs containing siPD-L1 (100 nM) were exposed to cells and incubated for an additional 2 h at 37 °C. After refreshed cell culture medium, the cells were incubated for another 24 h of proliferation,then the cells were collected and evaluated the content of ATP in the cells with ATP assay kit (Beyotime Biotechnology, China) under the instruction of the provider’s manual. The bioluminescence was detected using luminometer.

**Apoptosis of Tumor Cells**

To analyze cell apoptosis, 4T1 cells (5.0 × 10^5^ cells per well) were seeded into 6-well tissue culture plates. After 24 h proliferation, various NPs containing siPD-L1 (100 nM) were exposed to cells and incubated for an additional 2 h at 37 ^o^C. After refreshed cell culture medium, the cells were incubated for another 24 h of proliferation. The cells were collected and stained with Calcein-AM/PI and Annexin V-FITC Kit (Elabscience Biotechnology Co., Ltd, China), for 30 minutes in the absence of light. Subsequently, the stained cells were immediately analyzed using a flow cytometer and the data were processed using FlowJo software.

**In vitro Gene Silencing Effects**

For western-blotting analysis of PD-L1 protein expression level. Firstly, 4T1 cells (5.0 × 10^5^ cells per well) were seeded into 6-well tissue culture plates. After 24 h proliferation, various NPs containing siPD-L1 (100 nM) were exposed to cells and incubated for an additional 2 h at 37 ^o^C. After refreshed cell culture medium, the cells were incubated for another 48 h of proliferation. The cells were washed with pre-cooled PBS and then re-suspended in 200 μL RIPA lysis buffer supplemented with 1% proteinase inhibitor cocktail. The cell lysates were incubated on ice for 30 min and vortexed every 10 min. The total protein was gathered by centrifugation at 12,000 g for 30 min and the concentration was determined with BCA protein assay kit. Total protein (20 μg) was loaded on 10% sodium dodecyl sulfate polyacrylamide gel and electrophoresed at 80 mV for 30 min and 120 mV for 2 h. Then the proteins were transferred to polyvinylidene Fluoride (PVDF) membranes at 200 mA for 120 min then blocked with 5% skimmed milk on a horizontal shaker for 2 h. The membranes were incubated with the 1:2000 PD-L1 monoclonal antibody (Wuhan Sanying, China) overnight at 4 ^o^C followed by incubation with HRP-conjugated goat anti-mouse antibodies (1:2000 Zhongshan Goldenbridge Biotechnology Co. Ltd., Beijing, China) at room temperature for 2 h. Finally, the membranes were exposed using a ImagerQuant RT ECL System. GAPDH was used as endogenous control.

**In Vitro Induction of ICD**

4T1 cells (5.0 × 10^5^ cells per well) were seeded into 6-well tissue culture plates. After 24 h proliferation, various NPs containing siPD-L1 (100 nM) were exposed to cells and incubated for an additional 2 h at 37 ^o^C. After refreshed cell culture medium, the cells were incubated for another 48 h of proliferation. The cells were washed with pre-cooled PBS and then re-suspended in 200 μL RIPA lysis buffer supplemented with 1% proteinase inhibitor cocktail. The cell lysates were incubated on ice for 30 min and vortexed every 10 min. The total protein was gathered by centrifugation at 12,000 g for 30 min and was quantified using BCA protein assay kit to ensure consistency. Then, the total protein was levelled for HMGB-1 and CRT detection by ELISA kits (Mouse HMGB-1 ELISA Kit and Mouse CRT ELISA Kit),on the basis of the manufacturer’s instructions.

**In vivo distribution**

4T1 tumor models were prepared by subcutaneous injection of 2×10^6^ cells in the mammary fat pad of female BALB/c mice. When tumor size reached 300 mm^3^, different NPs (Cy7-labeled siRNA, the dose of 0.25 mg kg^-1^) were injected in the mice via tail vein. At the predetermined time points (6, 12, 24, 48 h), in vivo fluorescence distribution was visualized by the IVIS imaging system (Carestream Health, Rochester, NY, USA) with the excitation wavelength of 745 nm and the emission wavelength of 800 nm. Tumors and other organs, including heart, liver, spleen, lung and kidney, were excised and imaged.

**In Vivo Anti-tumor Study**

The anti-tumor studies were carried out in 4T1 tumor models. To establish the tumor-bearing mouse model, 6-week-old female BALB/c mice were subcutaneously injected with 2×10^6^ 4T1 cells in the mammary fat pad.When the tumor volume reached approximately 100 mm^3^, mice were injected intravenously with saline, MnP/siPD-L1 NPs, AHA@MnP/siNC NPs and AHA@MnP/siPD-L1 NPs (siRNA, the dose of 0.25 mg kg^-1^), every other day or two days for 4 times. The tumor volume was calculated by the following formula.

Tumor volume = (length × width^2^)/2

After the experiment, mice were sacrificed, and tumors were harvested for subsequent histopathology and tumor immune microenvironment (TIME) analyses. To evaluate the biocompatibility and toxicity of nanoparticles in vivo, the sera of tumor-bearing mice were obtained to evaluate liver function (AST, ALT) and renal function (CR, BUN) at the end of treatment. Meanwhile, the tumor and main organs (heart, liver, spleen, lung, and kidney) were collected from tumor-bearing mice for H&E staining.

For histopathology analysis and immunofluorometric assay, tumor tissues were excised at preset time points and cut into small pieces, which were treated additionally with collagenase type IV (1 mg mL^-1^) and DNase I (0.1 mg mL^-1^) under gentle shaking. After 30 min, the cell suspension was filtered through a 70-μm strainer. Cells were then stained with designated antibodies panels: Cy5-Anti-mouse CD11c Antibody, PC7-Anti-mouse CD8 Antibody, FITC-Anti-mouse CD4 Antibody, PE-Anti-mouse CD80 Antibody, APC-Anti-mouse Foxp3 Antibody, Cy5-Anti-mouse CD206 Antibody. Then it was imaged under optical microscopy. Moreover, the expression levels of PD-L1 in the 4T1 tumors were discovered through western blotting. ELISA kits were used to detect the concentrations of INF-γ and INF-α in serum and HMGB1 and CRT in tumour tissues.

**Bulk RNA Sequencing and Data Analysis**

RNA was extracted from tumor samples using Qiagen RNeasy Mini Kit. The isolated RNA of each sample was enriched for poly(A) templates and further used for whole mRNA-Seq on the Illumina Novaseq 6000 sequencer (Illumina) with PE150 reads.

The key steps of batch RNA sequence data analysis include: 1) using fastq (fqtools/v0.1.12) to filter the original data. 2) Download the reference genome and gene model annotation files from the genome website, build the reference genome index and compare the paired end clean reading with hisat2 v2.0.5. 3) Calculate the FPKM of each gene and the number of reads mapped to that gene. 4) The software DESeq2 (DESeq2/1.24.0) was used to analyze the differential expression between the two controls. Benjamin and Hochberg methods were used to adjust the generated p value to control the error detection rate. The genes with adjusted p value<=0.05 found by DESeq2 were assigned as differentially expressed genes. 5) The GO (clusterprofile/v3.18.1) enrichment analysis of differentially expressed genes and KEGG (clusterprofile/v3.18.1) pathway enrichment statistics were realized by using clusterprofiler software, and the go item and KEGG pathway with p<0.05 were set as significant enrichment results.

**Statistical analysis**

The results were expressed as mean ± standard deviation. For statistical analysis between two groups, student’s t-test for independent means was applied. Comparisons between multiple groups were made by one-way analysis of variation (ANOVA) followed by LSD multiple comparison test. Statistical analysis was performed using SPSS 16.0 software (SPSS Inc., Chicago). A value of *p* < 0.05 was considered as statistically significant.

**
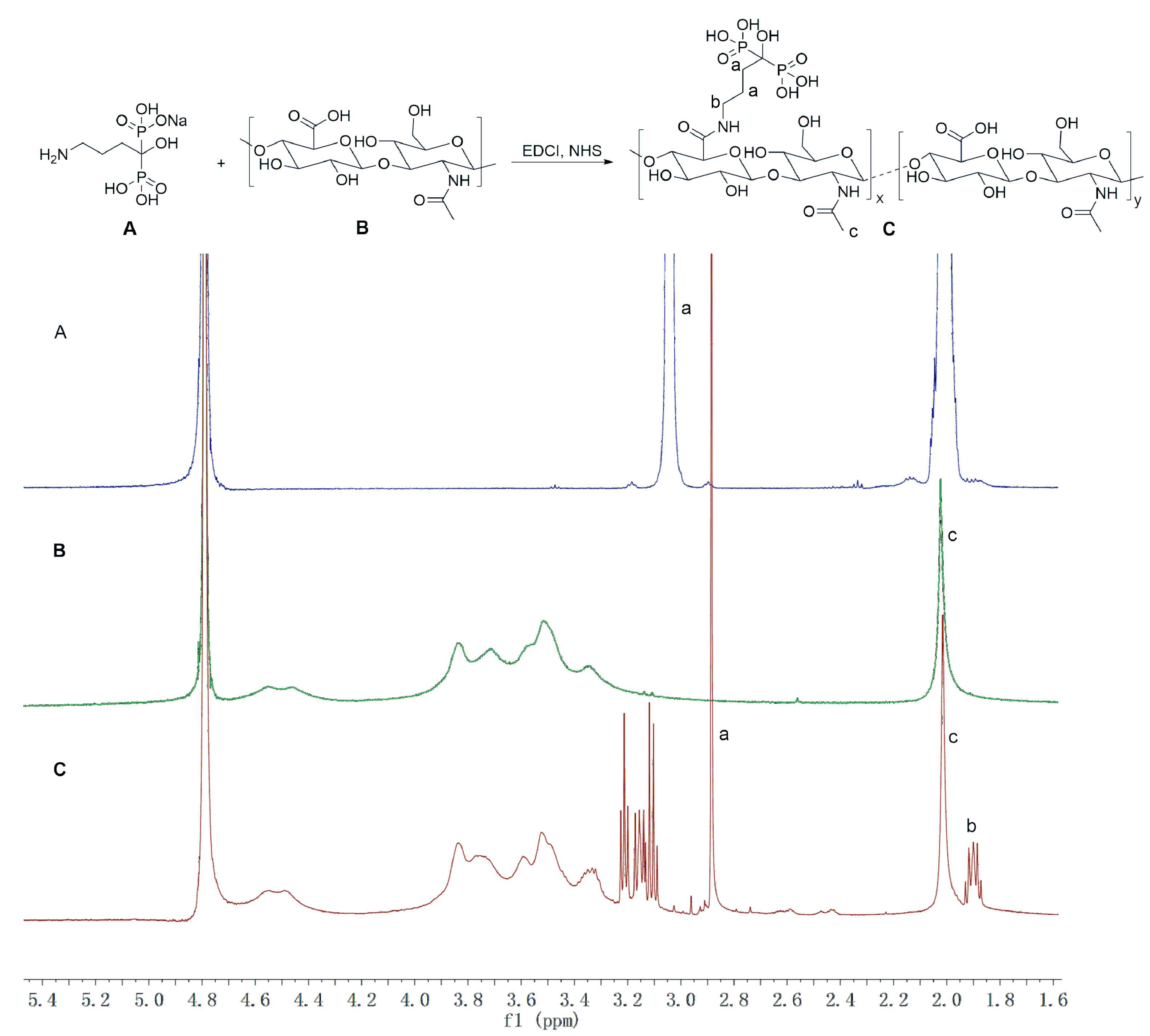
**

**Figure S1.** Synthesis procedure of alendronate-hyaluronan graft polymers (AHA) and the ^1^H-NMR analysis of HA and AHA.

**Table S1.** Size and polydispersity index (PDI) and zeta potential of nanoplexes (n = 3).

| Nanoplexes | Particle size  (d, nm) | Polydispersity  Index (PDI) | Zeta potential  (mV) | Encapsulation efficiency (%) |
| --- | --- | --- | --- | --- |
| AHA@MnP/siRNA | 196.13±0.55 | 0.21±0.015 | -17.25±1.01 | 91.3±2.99 |
| MnP/siRNA | 1899.67±79.20 | 0.17±0.046 | -10.61±0.60 | 89.2±1.34 |


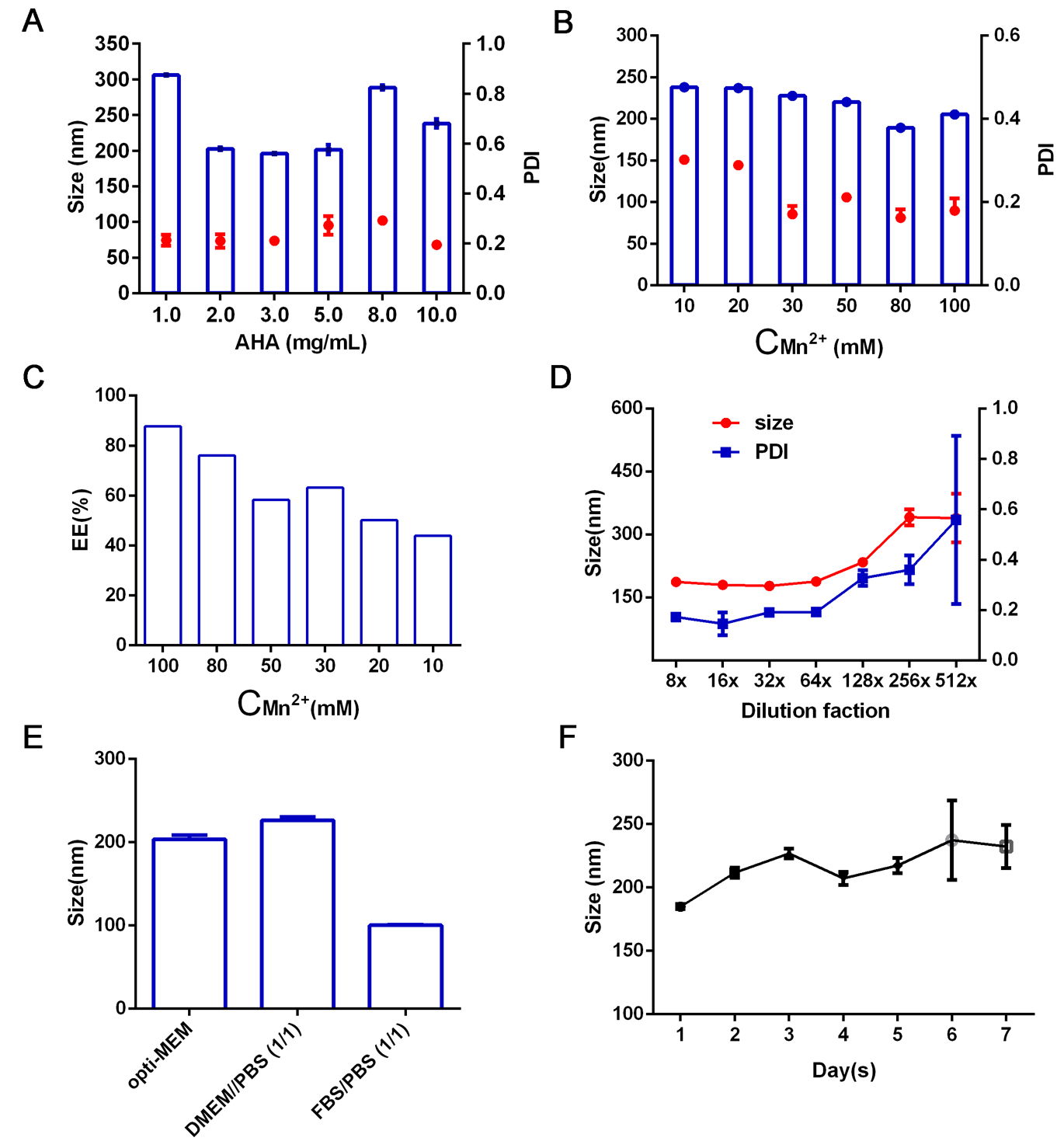


**Figure S2.** Formulation and prescription optimization of AHA@MnP/siPD-L1 NPs. DLS analysis on the size distribution and Polydispersity index (PDI) of AHA@MnP/siPD-L1 NPs with different concentration of AHA (A) and Mn^2+^ (B). (C) The siPD-L1 loading capability under different concentration of Mn^2+^ was detected by the Quant-iT™ RiboGreen® RNA Reagent and Kit (siRNA: 1 μM). (D) The diluted stability of optimised AHA@MnP/siPD-L1 NPs. (E) The changes of particle size in various solvent. (F) The changes of particle size for different time in room temperature. All the data were given as the mean ± SD (n=3).


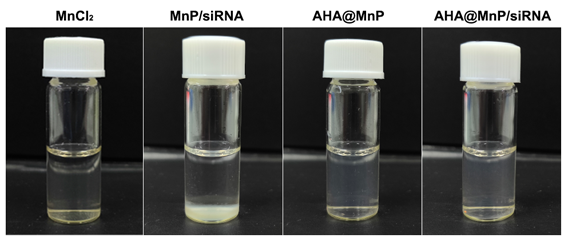


**Figure S3.** The picture of MnCl_2_ and various NPs.


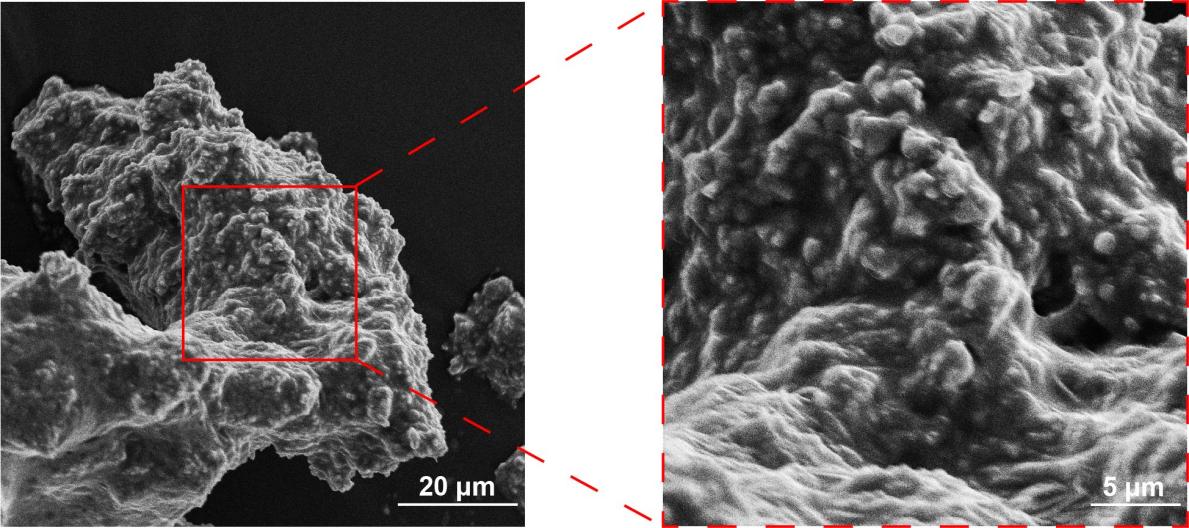


**Figure S4.** The SEM pictures of MnP/siRNA NPs


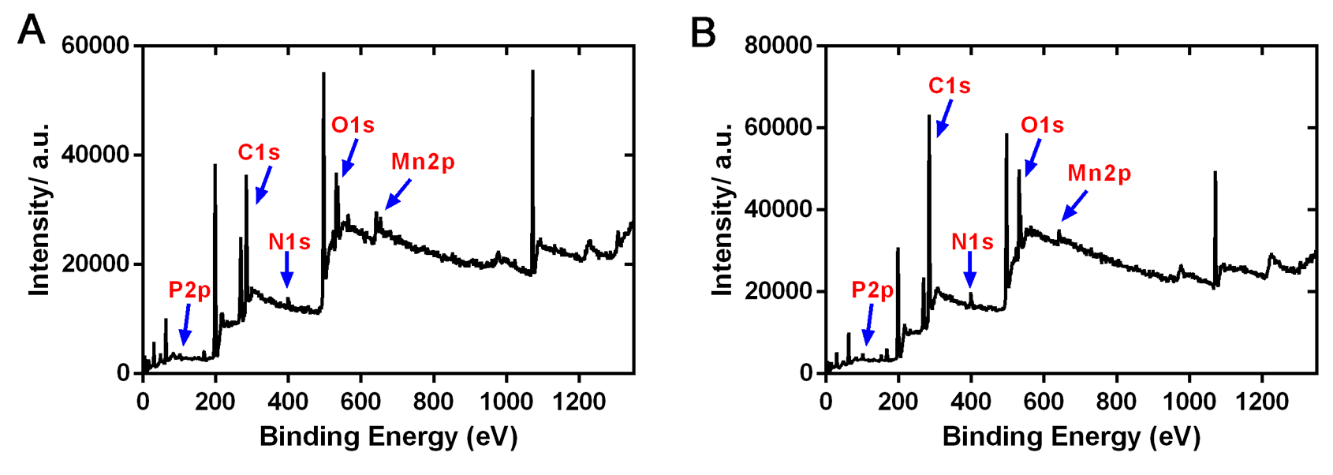


**Figure S5.** The XPS analysis of MnP/siRNA NPs (A) and AHA@MnP NPs (B).


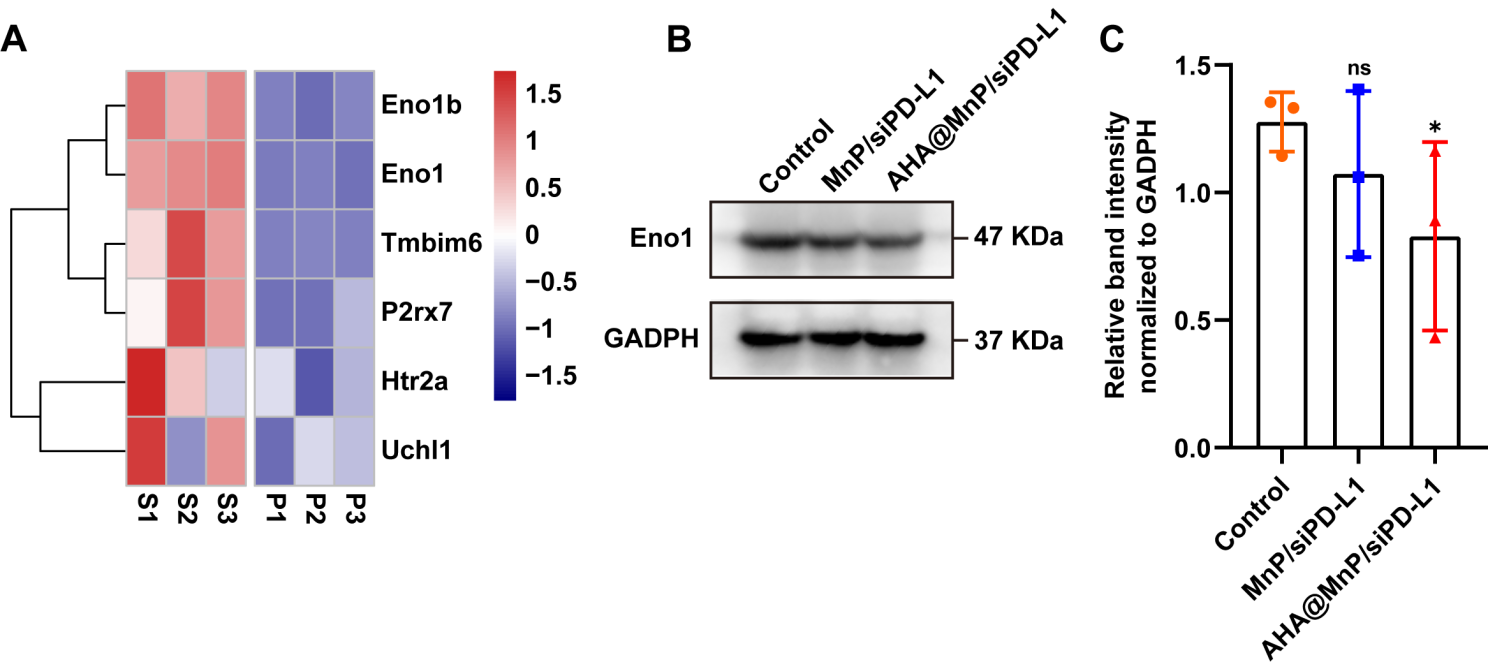


**Figure S6.** (A) Heatmaps depict the expression levels of anaerobic energy metabolism genes in the P and S groups. S group: Saline, P group: AHA@MnP/siPD-L1. (B) The expression of Eno1 protein for 4T1 cells treated with MnP/siPD-L1 or AHA@MnP/siPD-L1 NPs; (C) Quantitative analysis the protein intensity rate of 4T1 cells. Data are presented as the mean ± SD (n=3)


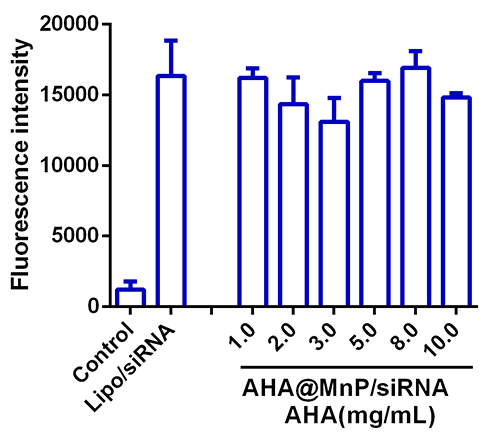


**Figure S7.** Quantitative analysis of intracellular fluorescence intensities of AHA@MnP/siRNA by flow cytometry (FAM-labeled siRNA: 100 nM, n=3).


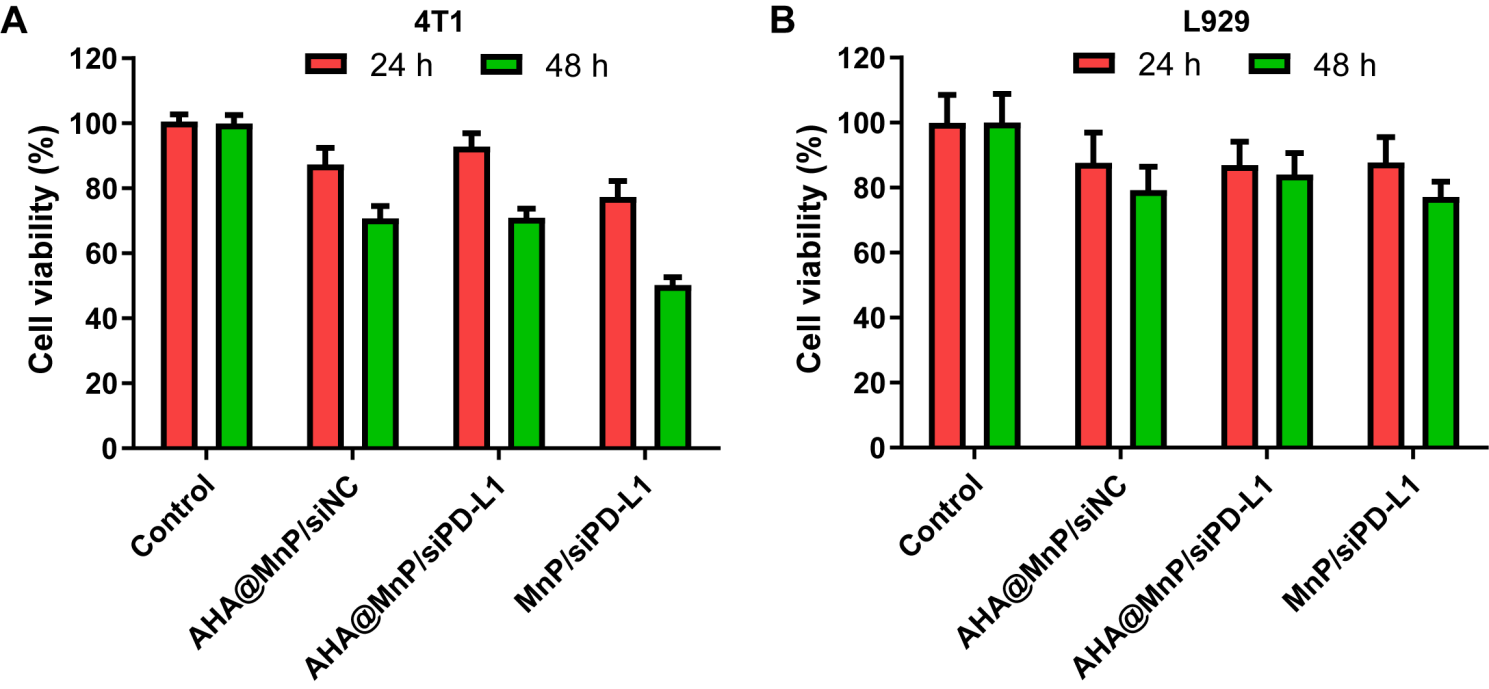


**Figure S8.** The cell viabilities of 4T1 and L929 cells after treatment with different nanoparticles for 24 h and 48 h (n = 6).


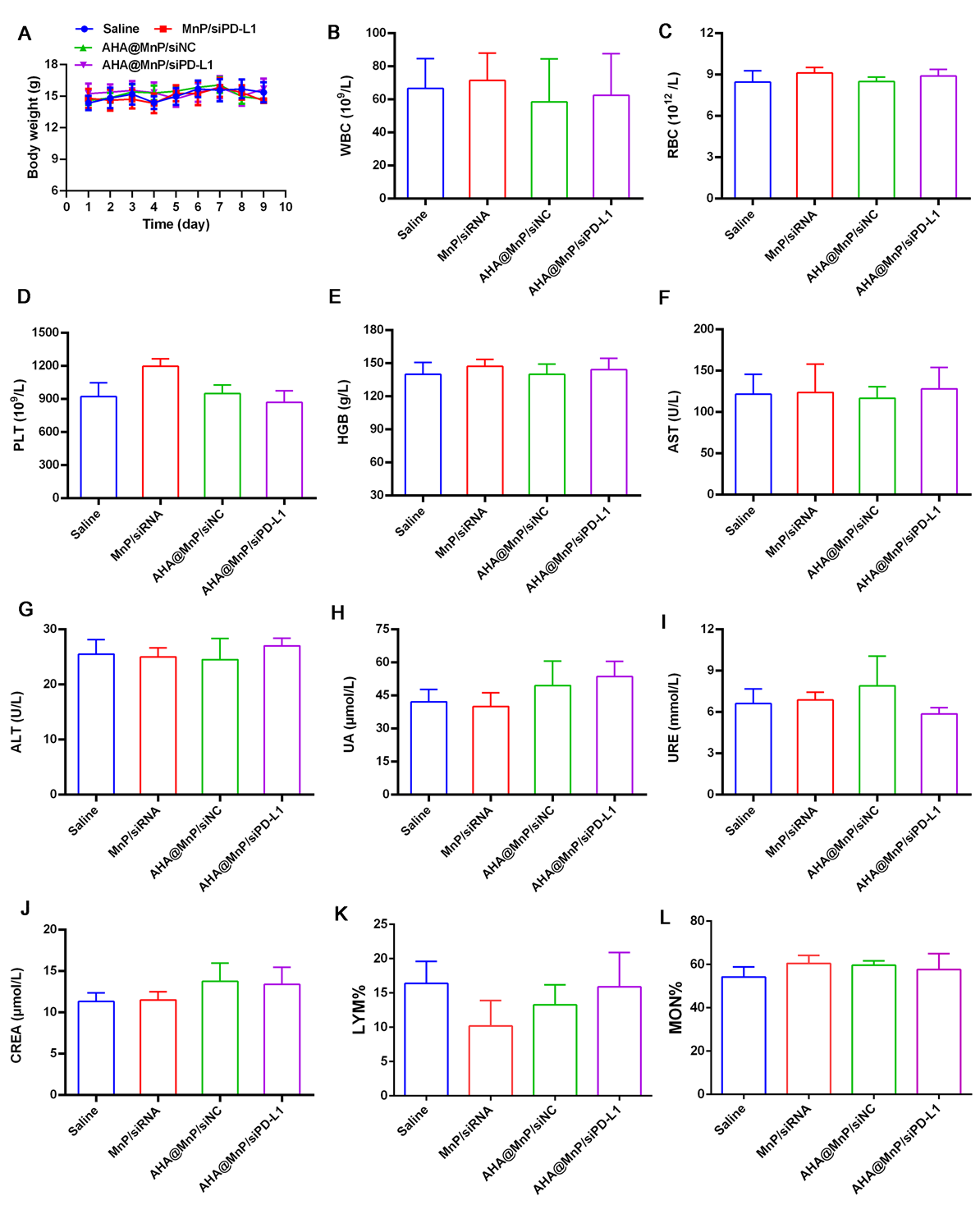


**Figure S9.** The in vivo safety study in BALB/c mice bearing 4T1 cancer cells after administration of different NPs. (A) The body weight change in the process of NPs treatment. (B~L) The blood routine and biochemical analysis after administration of different NPs. WBC: white blood cell, RBC: red blood cell, PLT: platelet, HGB: hemoglobin, AST: aspartate aminotransferase, ALT: alanine aminotransferase, UA: uric acid, CREA: creatinine, LYM: lymphocyte, MON: monocytes.


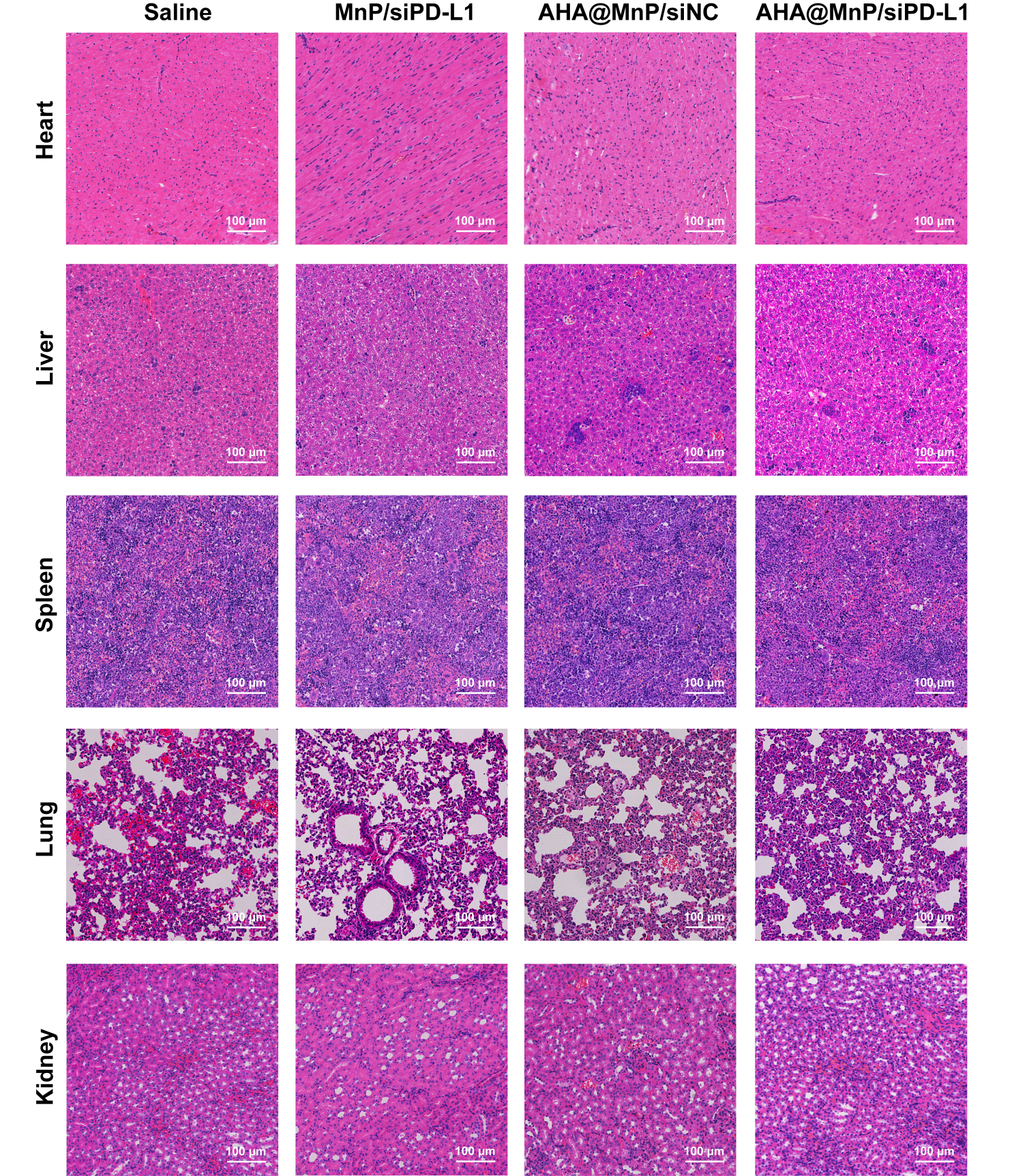


**Figure S10.** Haematoxylin-eosin staining of tissue sections. The major organs were taken from the sacrificed BALB/c mice bearing 4T1 cancer cells at the end of drug administration. Scale bar: 100 μm


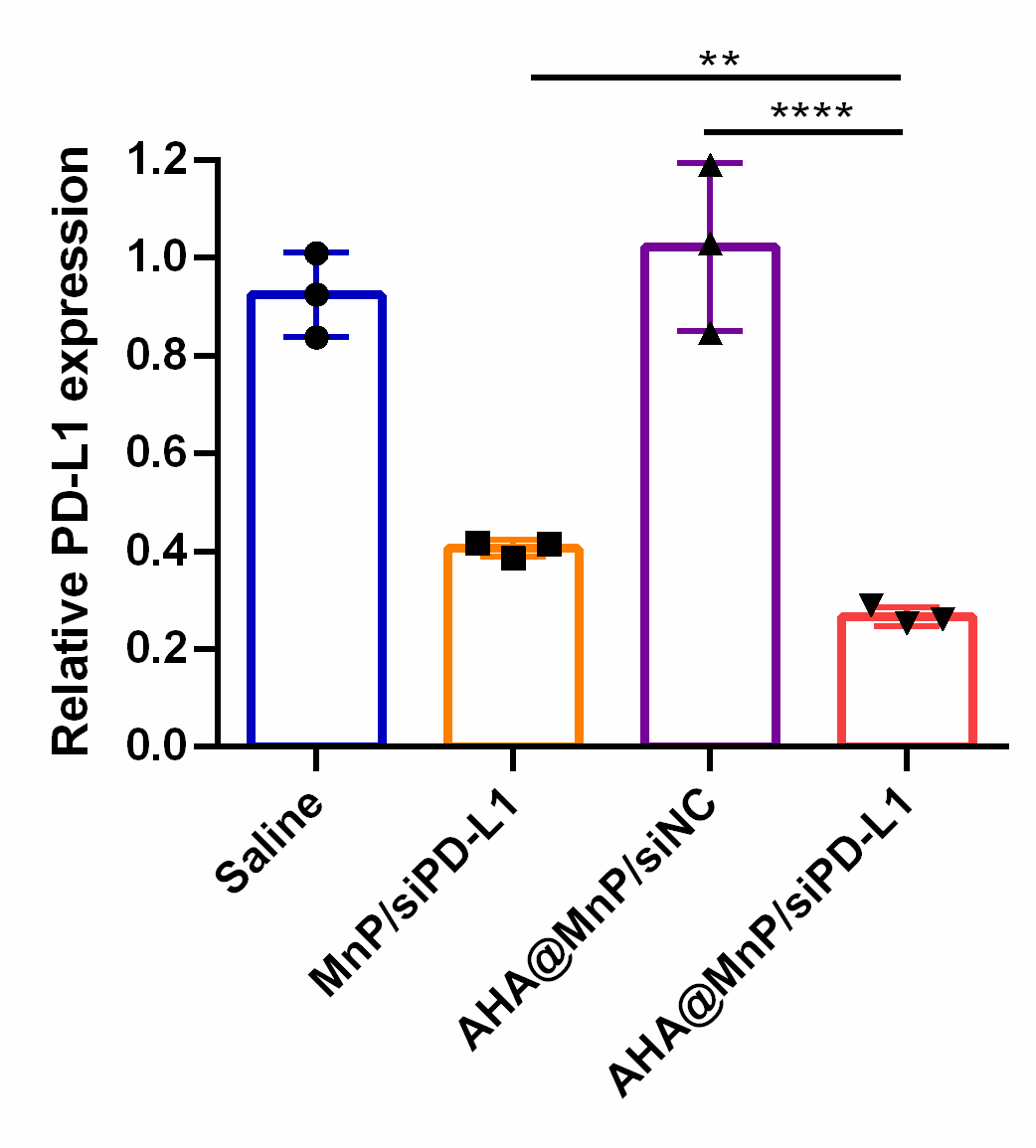


**Figure S11.** The expression of the PD-L1 protein in tumor detected by the western-blot assay. The data are shown as mean ± SD (n=3). **p < 0.01 and ****p < 0.0001.
